# Multiplex engineering using microRNA-mediated gene silencing in CAR T cells

**DOI:** 10.3389/fimmu.2025.1647433

**Published:** 2025-08-21

**Authors:** Giulia Golinelli, John Scholler, Audrey Roussel-Gervais, Antonija Šakić, Sten Ilmjärv, Decheng Song, Khatuna Gabunia, Mei Ji, Ting J. Fan, Aasha Gupta, Mansi Deshmukh, Abdulla Berjis, Riccardo Cuoghi Costantini, Kimberly Apodaca, Neil C. Sheppard, Sven Kili, Massimo Dominici, Marco Alessandrini, Carl H. June, Bruce L. Levine

**Affiliations:** ^1^ Center for Cellular Immunotherapies, Perelman School of Medicine, University of Pennsylvania, Philadelphia, PA, United States; ^2^ Laboratory of Cellular Therapy, Division of Oncology, Department of Medical and Surgical Sciences for Children and Adults, University-Hospital of Modena and Reggio Emilia, Modena, Italy; ^3^ Antion Biosciences SA, Geneva, Switzerland; ^4^ School of Engineering and Applied Science, University of Pennsylvania, Philadelphia, PA, United States; ^5^ Unit of Clinical Statistics, University Hospital of Modena, Modena, Italy; ^6^ Department of Pathology and Laboratory Medicine, University of Pennsylvania, Philadelphia, PA, United States; ^7^ Parker Institute for Cancer Immunotherapy at University of Pennsylvania, University of Pennsylvania, Philadelphia, PA, United States

**Keywords:** CAR T, miRNAs, CRISPR/Cas9, multiplexing, solid tumors, gene silencing

## Abstract

**Background:**

Multiplex gene-edited chimeric antigen receptor (CAR) T-cell therapies face significant challenges, including potential oncogenic risks associated with double-strand DNA breaks. Targeted microRNAs (miRNAs) may provide a safer, functional, and tunable alternative for gene silencing without the need for DNA editing.

**Methods:**

As a proof of concept for multiplex gene silencing, we employed an optimized miRNA backbone and gene architecture to silence T-cell receptor (TCR) and major histocompatibility complex class I (MHC-I) in mesothelin-directed CAR (M5CAR) T cells. The efficacy of this approach was compared to CD3ζ and β2-microglobulin (β2M) CRISPR/Cas9 knockout (KO) cells. miRNA-expressing cassettes were incorporated into M5CAR lentiviral vectors, enabling combined gene silencing and CAR expression. Antitumor activity was evaluated using *in vitro* assays and *in vivo* pancreatic ductal adenocarcinoma models.

**Results:**

Silenced (S) M5CAR T cells retained antitumor functionality comparable to, and in some cases exceeding, that of KO cells. *In vivo*, S M5CAR T cells achieved tumor control with higher persistence and superior metastasis prevention. *In vitro* assays demonstrated enhanced resistance to alloreactive natural killer (NK) cells and peripheral blood mononuclear cells (PBMCs).

**Conclusions:**

Titratable multiplex gene silencing via targeted miRNAs offers an alternative to gene editing for CAR T cells, with potential advantages in potency, persistence, metastasis prevention, and immune evasion for allogeneic products. This strategy may overcome tumor-induced immunosuppression while avoiding the risks associated with DNA double-strand breaks.

## Introduction

The development of chimeric antigen receptor (CAR) T cell therapies has revolutionized cancer treatment, particularly for hematological malignancies. However, challenges persist in optimizing efficacy and accessibility without compromising safety (1). Factors such as patient age, prior treatments, and the tumor microenvironment (TME) can lead to dysfunctional and exhausted T cells, hindering the consistency, potency, and durability of CAR T cell products (2–4).

To enhance CAR T cell efficacy, genetic engineering strategies have been employed to render these cells resistant to inhibitory signals within the TME. Additionally, engineering CAR T cells from healthy donors aims to mitigate alloreactivity and shield these T cells, that have not been compromised by the effects of chemotherapy and disease, from immune rejection in recipients through targeted genetic modifications (5). Several genome-editing strategies have been applied for this purpose, including early tools such as transcription activator-like effector nucleases (TALENs), zinc finger nucleases, and CRISPR/nucleases (5). However, multiplexed gene editing with nucleases can result in aneuploidy, chromosomal translocations, and p53-mediated genotoxicity associated with DNA double-strand breaks (6–8). Moreover, these technologies often produce heterogeneous editing outcomes due to competing DNA repair pathways (9).

Base editing (BE) and prime editing (PE) offer more precise genetic modifications by reducing reliance on DNA double-strand breaks. ‘Off-the-shelf’ allogeneic and multiplex base-edited CAR T cells targeting CD7 are under investigation (10). However, BE and PE have been shown to trigger adverse transcriptional responses and still produce DNA double-strand breaks, deletions, and translocations in edited human hematopoietic stem/progenitor cells, albeit at a lower frequency than Cas9 (11). Furthermore, genome editing is frequently introduced as a secondary step in the manufacturing process via nucleofection (NF) or electroporation (EP), reducing cell viability and yield.

MicroRNAs (miRNAs) are key regulators of gene expression across mammalian cell types, functioning post-transcriptionally by binding messenger RNA (mRNA) to suppress protein translation through targeted repression or mRNA cleavage. While naturally occurring miRNAs often silence multiple targets within a cell, artificially designed miRNAs can effectively silence specific genes of interest with high efficiency and accuracy. By employing an optimized miRNA backbone (mirGE) and gene architecture for high targeting fidelity (12–14), multiple distinct miRNAs can be cloned within a single viral construct, referred to as multiplexed miRNAs, to achieve functional silencing of different target genes without the safety risks and inefficiencies associated with DNA editing.

The mirGE backbone is a miRNA mimic that preserves canonical miRNA processing while being specifically optimized to enhance target-specific silencing and minimize off-target effects. This optimization includes exclusive target sequence design for a gene of interest, improved processing accuracy of the miRNA, and preferential use of the guide strand for targeted silencing. As a result, mirGE-mediated gene silencing is highly efficient, with increased on-target activity and reduced unintended effects arising from imprecise guide strand processing or collateral silencing by the complementary passenger strand (12, 14). To facilitate more efficient gene transfer and multi-target silencing, the gene architecture driving mirGE expression was intentionally miniaturized and optimized to enable multiplexing of the miRNAs (14). Notably, these improvements enable the development of multiplexed miRNA constructs using a single, unmodified mirGE backbone, avoiding the structural complexity and potential processing variability associated with chimeric cluster designs based on multiple endogenous scaffolds (15).

This study investigates the efficacy of multiplex gene silencing in healthy donor CAR T cells within an aggressive pancreatic ductal adenocarcinoma (PDAC) model. As a proof of concept, we designed miRNAs to target the transcripts of the CD3ζ and β2-microglobulin (β2M) subunits of the T-cell receptor (TCR) and major histocompatibility complex class I (MHC-I) protein complexes, respectively. We compared miRNA-mediated silencing to CRISPR/Cas9-based gene editing in developing non-alloreactive and hypoimmunogenic CAR T-cell products. Our objectives included eliminating TCR expression and finely tuning MHC-I silencing, thereby preventing graft-versus-host disease (GvHD) and ensuring balanced protection from host CD8 T cell- and natural killer (NK) cell-mediated immune rejection. We developed lentiviral vectors to transfer bimodal gene constructs expressing both CAR and miRNA gene silencing cassettes. This multiplex gene engineering strategy enables uniform gene silencing and CAR expression across modified primary human T cells through a single gene augmentation step. By avoiding the induction of DNA double-strand breaks, this gene-silencing approach may help overcome barriers to the clinical development of CAR T cells for allogeneic applications and enhance the potency of both allogeneic and autologous immune effector cells.

## Materials and methods

### General cell culture

AsPC-1 cell line was obtained from American Type Culture Collection (ATCC) and was cultured in R20 culture media [RPMI 1640 (Gibco) + 20% FBS (Avantor Seradigm, 97068-086), 1% penicillin/streptomycin (50 IU/mL; Gibco), 1% GlutaMAX™ (Gibco), 2% 1M Hepes (Gibco)] at 37°C in 5% CO_2_. AsPC-1-click beetle green (CBG) and green fluorescent protein (GFP) expressing cells (AsPC-1 CBG GFP cells) were generated by lentiviral transduction for cell killing assays and *in vivo* studies. The cell lines were selected by sorting on FACS Aria Sorter (BD Biosciences) and monitored for growth and stable gene expression for 8 weeks. All cell lines were authenticated by the University of Arizona Genetics Core and were tested for the presence of mycoplasma contamination (MycoAlert^®^ Mycoplasma Detection Kit, Lonza) every 6 months and before being used *in vivo*.

### CRISPR/Cas9-guide design and screening

CRISPR single guide RNA (sgRNA) targeting CD3ζ were designed using multiple softwares: Benchling, CRISPick (Broad Institute), IDT, ChopChop. The most common sgRNAs were selected and chemically modified and synthesized by Integrated DNA Technologies (IDT) (**Supplementary Table 1**).

One of five sgRNA targeting CD3ζ (sgRNA_4) was selected for further experiments after validation for highest deletion efficiency at the DNA and protein levels. β*2M* sgRNA sequence was taken from the product designed for NCT05037669, while the TRAC sgRNA sequence was taken from Ren et al. (16), both in **Supplementary Table 1**. A lentiviral vector for the expression of the second generation anti-CD19 CAR (CAR19) composed of the FMC63 scFv and CD8α hinge and transmembrane domains fused to 4-1BB and CD3-ζ cytoplasmic signaling domains expressed under control of the EF-1α promoter (17) was also employed for CD3ζ and/or β2M deletion evaluation on CAR T cells.

### Design and screening of miRNA silencing constructs

miRNA target sequences for CD3ζ and β2M (**Supplementary Table 2**) were previously designed using the methodology described in Myburgh et al. (12) as well as online resources for target sequence design based on sequence prioritization, and individual scoring of each target sequence to identify the potentially best performing candidates, while mitigating the risk of potential off-target gene silencing. Prioritized target sequences were synthesized within a mirGE backbone (12) by Thermo Fisher Scientific and subsequently cloned into a pENTR plasmid using basic restriction enzymes. The final lentivector plasmid was generated by an LR Clonase II (Invitrogen, Carlsbad, CA)-mediated recombination of a pENTR plasmid containing an elongation factor 1 short promoter (pENTR-L4-EFs-L1R), a pENTR plasmid containing the mirGE expression cassette (pENTR-L1-spacer-mirGE-L2), and a pCWX-R4-DEST-R2-PGK-mCherry lentivector destination plasmid. Successful cloning of all constructs was confirmed via restriction enzyme digestion pattern and DNA sequencing. Silencing lentiviral vectors were then used to transduce T cells derived from healthy donors. Transduction efficiency (based on mCherry reporter gene expression) and target silencing were evaluated by flow cytometry.

### Lentiviral vector design

A bidirectional dual-promoter expression system was selected for optimal simultaneous gene silencing and CAR expression. Optimized miRNA expression constructs for silencing of CD3ζ and/or β2M, driven by the EFs promoter, were cloned into the pTRPG vector, which encodes M5CAR under the control of the human phosphoglycerate kinase (hPGK) promoter, in a bidirectional promoter configuration. The second generation M5CAR is composed of a human Msln-binding (M5) scFv and CD8α hinge and transmembrane domains fused to 4-1BB and CD3ζ cytoplasmic signaling domains expressed under control of the PGK promoter. The M5CAR expression construct was combined with a scrambled miRNA target sequence in scrambled M5 CAR T cells, miRNAs for single silencing of CD3ζ and β2M transcripts in S CD3ζ M5 CAR T cells and sβ2M M5 CAR T cells, respectively, and miRNAs for dual silencing of CD3ζ and β2M in S CD3ζ&β2M M5CAR T cells.

### Lentiviral vector production

Replication defective lentiviral vectors were generated by transient transfection of HEK293T cells (ATCC ACS-4500) using Lipofectamine 2000 (ThermoFisher Scientific, Cat#11668500). Approximately 10 x 10^6^ cells were plated in T150 culture vessels in R10 culture media (RPMI 1640 + 10% FBS + 1% penicillin/streptomycin (50 IU/mL), 1% GlutaMAX™, 2% 1M Hepes) and incubated overnight (o/n) at 37°C. 18–24 hours later, cells were transfected using a combination of Lipofectamine 2000 (96mL, Invitrogen), pTRP gag/pol (Lot# RR24JUN21D-2) (18µg), pTRP RSV-Rev (Lot# RR24JUN21B) (18µg), pTRP Cocal (Lot# RR24JUN21A-2) (7µg) packaging plasmids and 15µg of expression plasmid (anti-Msln M5bbz scFv cloned in pTRPG). Lipofectamine and plasmid DNA were diluted in 3 mL Opti-MEM media (Gibco, Life Technologies) prior to transferring into lentiviral production flasks. At both 24 and 48 hours following transfection, culture media was isolated and concentrated using high-speed ultracentrifugation (8000g o/n and then 25,000g for 2.5 hours). To generate the lentiviral stocks, the resulting concentrated lentivirus batches were resuspended in cold R10 media and stored at -80C.

### CAR T cell manufacturing

CAR T cells were manufactured from frozen human healthy donor (ND) bulk T cells or peripheral blood mononuclear cells (PBMCs). In both cases, PBMCs were acquired through leukapheresis from healthy volunteers by the University of Pennsylvania Human Immunology Core (HIC), with the approval of the University’s Institutional Review Board. Study participants provided written informed consent, in compliance with the Declaration of Helsinki, the International Conference on Harmonization’s Good Clinical Practice guidelines, and the United States Common Rule. Lymphoprep™ (Stemcell Technologies) followed by the RosetteSep™ Human T Cell Enrichment Cocktail (Stemcell Technologies) were used by the HIC for PBMC and bulk T cell isolation, respectively. From PBMCs, CD4 and CD8 T cells were separately isolated by the HIC using CD4 (Catalog# 15062; Stemcell Technologies) and CD8 (Catalog# 15063, Stemcell Technologies) selection kits, then pooled. Cells were cultured in T cell media composed by the OpTmizer™ CTS™ T-Cell Expansion SFM (Thermo Fisher), 5% human AB serum (Valley Biomedical), 1% penicillin/streptomycin (50 IU/mL), and 1% GlutaMAX™.

Cells undergoing miRNA-mediated gene silencing were activated using CTS™ Dynabeads™ CD3/CD28 (Catalog# 40203D; Thermo Fisher) at a 3:1 bead-to-cell ratio and resuspended at 1 x 10^6^ cell/mL in T cell media (day 0). Single lentiviral vectors coding for the M5CAR (3) and CD3ζ and/or β2M-targeting miRNAs were used for transduction approximately 24 hours post bead stimulation (day 1) at an MOI of 3. For each silencing condition, 2 x 10^6^ cells were transduced, as in the control M5CAR T cell group.

Cells undergoing the CRISPR/Cas9-genome engineering were incubated o/n at 5 x 10^6^ cells/mL with 10 ng/mL IL-7 and IL-15 (Catalog# 200–07 and 200-15, respectively; Peprotech) (day -1). CRISPR/Cas9-gene deletion, T cell activation, transduction, expansion, and KO validation were performed following an optimized protocol previously described (18). Approximately 8–12 x 10^6^ cells were used as the starting amount for each condition. Briefly, primary T cells were nucleofected (day 0) using the Lonza 4D-Nucleofector Core/X Unit and the P3 Primary cell 4D-nucleofactor X Kit (Lonza). For Cas9 and sgRNA delivery, the ribonucleoprotein (RNP) complex was formed by incubating 10mg of Spy Fi Cas9 (Catalog# 9214; Aldeveron) with 5mg of sgRNA (IDT) for 10 minutes at room temperature (RT). Cells were resuspended at a concentration of 8–12 x 10^6^ cells/100µL in the specified kit buffer (P3 Solution with Supplement). The RNP complex, 100µL of resuspended cells, and 4.2 µL of 4 µM IDT Electroporation Enhancer (a non-homologous ssDNA oligonucleotide) were combined and nucleofected in a cuvette. Pulse code EH115 was used for primary T cells. After nucleofection (NF), the cells were incubated in T cell media at 37°C in 12-well plates. Four hours later, nucleofected cells were then activated using CTS™ Dynabeads™ CD3/CD28 at a 3:1 bead-to-cell ratio and resuspended in T cell media at a concentration of 2 x 10^6^ cells/mL. 24 hours later, for each NF condition, cells were counted and the M5CAR lentiviral vector was used for transduction at an MOI of 3 (day 1). For each KO condition, all counted cells were transduced.

Over both protocols, beads were removed on day 5 after stimulation. Cells were then monitored daily using the Multisizer 4 Coulter Counter (Beckman Coulter) until growth kinetics and cell size demonstrated that they had rested from stimulation. On average, T cells were grown for 9–10 days in the presence of 10 ng/mL of IL-7 and IL-15 and maintained at 8 x 10^5^ cells/mL prior to cryopreservation. A proportion of cells in groups with either CD3ζ S or KO, or dual CD3ζ and β2M S or KO, were depleted of TCRαβ before cryopreservation using the EasySep™ Human TCR Alpha/Beta Depletion Kit (Stemcell Technologies). Fold expansion was expressed as the ratio of the final number of cells to the initial number of cells, while population doublings (PDs) were calculated as the log_2_ of this ratio. The percentage reduction in T cell numbers after NF was calculated as the difference between pre- and post-NF cell counts, divided by the pre-NF count and multiplied by 100. At the end of expansion, cells were collected, and flow cytometry analysis was performed for evaluation of M5CAR+ T cell percentage, CD3ζ and/or β2M silencing or KO on T cells, memory phenotype by CD45RO and CCR7, and activation marker expression (4-1BB, TIGIT, PD-1, TIM-3, LAG-3, and OX40). Prior to all experiments, T cells were thawed and rested at 2 x 10^6^ cells/mL in T cell media at 37°C for 16 hours.

### Genomic DNA extraction, Sanger sequencing, indel detection

From each T cell culture engineered with CRISPR/Cas9, 3 x 10^6–^5 x 10^6^ cells were flash frozen as dry cell pellets at the end of expansion. At time of DNA extraction, cell pellets were thawed and resuspended in 200µl Phosphate Buffer Saline (PBS). Genomic DNA from nucleofected cells was isolated using the DNeasy Blood & Tissue Kit (Catalog# 69504; Qiagen) and 200 ng DNA was PCR amplified using AccuPrime™ Pfx SuperMix (Catalog# 12344040; Thermo Fisher) and 10µM forward and reverse primers flanking the region of interest. Primers were designed such that the amplicon was at a target size of 600-700bp. PCR products were gel purified, Sanger sequenced, and trace files were analyzed using both TIDE (Tracking of Indels by Decomposition; Netherlands Cancer Institute) (19) and ICE (Inference of CRISPR Edits; Synthego) (20) analyses to detect deletion efficiency at the genomic level. PCR primers and sequencing primers were designed to detect each target locus. The following primers for target sequence amplification (‘PCR’) and Sanger sequencing (‘Seq’) were used for validation of deletion efficiency: CD3ζ.PCR.F 5’ CCATTGCCCCAGGTTCTTTG 3’, CD3ζ.PCR.R 5’ AAGCCCCTTTGTCACCAGTA 3’, CD3ζ.Seq.F 5’ CCACATCTGCCGTTGGTG 3’, CD3ζ.Seq.R 5’ TTCCCTGGGACACTCTGATG 3’; β2M.PCR.F 5’ TCCAGCCTGAAGTCCTAGAATG 3’, β2M.PCR.R 5’ TTATCGACGCCCTAAACTTTGT 3’, β2M.Seq.F 5’ ACAGACAGCAAACTCACCCA 3’, and β2M.Seq.R 5’ CCAAAGGTCTCCCCTGCTC 3’.

### Bioluminescence-based cytotoxicity co-culture assays

AsPC-1 CBG GFP target cells were seeded in white 96 well plates at 4000 cells/well. After 24 hours, M5CAR+ T cells were added at different effector:target (E:T) ratios (1:3, 1:1, 3:1, 10:1 E:T ratios). M5CAR T cell groups were matched for total T-cell dose added in co-culture by not transduced (NTD) normalization. Target cell survival was measured using bioluminescent quantification. After 24, 48 hours, and 4 days, D-luciferin potassium salt (Perkin-Elmer) was added to cell cultures at a final concentration of 150μg/mL and incubated at 37°C for 5 minutes. Bioluminescent signal was detected using the BioTek Synergy H4 plate reader, and the signal was analyzed using the BioTek Gen5 software. Tumor cells containing the medium alone or treated with cell lysis buffer (Qiagen) were used as controls, representing 100% and 0% cell viability, respectively. Triplicate wells were averaged, and cell viability was calculated with the following formula: % cell viability = 100 × (experimental bioluminescence − 0% bioluminescence)/(100% bioluminescence  − 0% bioluminescence).

### Real-time cytotoxicity co-culture assays

Using the same plate settings as for the bioluminescence-based co-culture assays, AsPC-1 CBG GFP viability was monitored in real-time using the IncuCyte^®^ SX5 (Sartorius) over 6 days. The amount of live GFP+ tumor cells for each condition was quantified by the IncuCyte SX5 live imaging system. For data analysis, the fluorescence integrated intensity per well were normalized to the time point of CAR T cell addition (0 hours) and triplicate wells were averaged. On day 6, cells were collected, and flow cytometry analysis was performed for evaluation of M5CAR+ T cell absolute number and percentage over total T cells, CD3ζ and/or β2M silencing or KO on T cells, memory phenotype by CD45RO and CCR7, and activation markers (4-1BB, TIGIT, PD-1, TIM-3, LAG-3, and OX40). CountBright™ Absolute Counting Beads (Catalog# C36950; ThermoFisher) were used as an internal standard according to the manufacturer’s instructions to calculate absolute cell counts in cell suspensions.

### NK-cell killing assays

Primary non-matched healthy donor NK cells were enriched by the HIC from PBMCs using the RosetteSep™ Human NK Cell Enrichment Cocktail (Stemcell Technologies). Purified NK cells were cultured o/n in T cell media supplemented with IL-2 cytokine. TCRαβ-depleted M5CAR T cells were stained with 1 μM CalceinAM (BioLegend) and used as target cells. These NK cells were then mixed with the M5CAR T cells at NK:T ratios of 12:1 and 6:1. After 4 hours of co-culture, cells were harvested and stained with 3 µM propidium iodide (PI; ThermoFisher). The percentage of viable target cells (PI-negative/CalceinAM^high^-positive) was assessed via flow cytometry. For baseline determination, target cells stained with CalceinAM and maintained in culture for 4 hours were mixed with NK cells at a 1:1 NK:T ratio just before final staining and subsequent flow cytometry determination of the percentage of viable target cells (baseline). This baseline value was used to normalize the co-culture values. The data were expressed as the ratio of the percentage of viable target cells in the co-culture condition to the percentage of viable target cells at baseline.

### One-way mixed lymphocyte reaction assay

Donor TCRαβ-depleted M5CAR T cells (stimulator) were co-cultured with non-matched healthy donor PBMCs (responders) at a 1:1 E:T ratio for six days. NK cells were kept or removed from PBMCs using human CD56 MicroBeads and LD columns (Miltenyi Biotech) or the EasySep™ Human NK Cell Isolation Kit (Stemcell Technologies), as per manufacturer’s instruction. Stimulator TCRαβ-depleted M5CAR T cells were irradiated at 2500 cGy. T cell media was added after three days. PBMC proliferation was determined by tritiated thymidine (^3^H-TdR, 100µCi/mL, PerkinElmer) incorporation for the last 8 hours of culture. Data were analyzed by the Microbeta2 Lumijet microplate reader (PerkinElmer).

### Flow cytometry and sorting

For flow cytometry and sorting assays cells were stained in fluorescence-activated cell sorting (FACS) buffer consisting of PBS (Gibco) and 2% fetal bovine serum (ThermoFisher). In some assays, CountBright™ Absolute Counting Beads (ThermoFisher) were used to calculate absolute cell counts. A specific cell population was considered detectable and reliably quantifiable when at least 30 events were recorded under the established experimental acquisition conditions. Antibodies specific for human CD45 (Catalog# 368524, Clone 2D1), CD4 (317414, OKT4), CD8 (344721, SK1), TCRαβ (306714, IP26), CD3ϵ (300400/300429 UCHT1), MHC-I (311436, W6/32), β2M (316316/316304, 2M2), CCR7 (353244/353244, G043H7), CD45RO (304231, UCHLI), OX40 (350021, Ber-ACT35), 4-1BB (309827, 4B4-1), PD-1 (329927, EH12.2H7), TIM-3 (119721, RMT3-23), and TIGIT (372735, A15153G), were purchased from BioLegend. The anti-human CD8 antibody was obtained from BD Pharmigen (Catalog# 560179) The anti-human LAG-3 antibody (Catalog# 61-2239-42) was purchased from eBioscience. M5CAR was detected using the Biotin-SP (long spacer) AffiniPure™ F(ab’)_2_ Fragment Goat Anti-Human IgG, F(ab’)_2_ fragment specific (Catalog# 109-066-006; Jackson ImmunoResearch), followed by a PE-streptavidin (Catalog# 554061, BD Biosciences). Cell viability was measured using LIVE/DEAD™ Fixable Violet Dead Cell Stain Kit (Catalog# L34964; Thermo Fisher) according to the manufacturer’s instructions. Samples were acquired on an LSRII Fortessa Cytometer (BD Biosciences) or FACSymphony A5 SE Cell Analyzer (BD Biosciences). All data analysis was performed using FlowJo 9.0 software (FlowJo, LLC). Sorting assays were performed using a FACS Aria Cytometer (BD Bioscience). At the end of expansion, levels of target KO and silencing were assessed via flow cytometry. Of note, CD3ϵ, TCRαβ, and β2M expression was gated based on the control M5CAR T cells. For KO conditions, we determined the percentage of cells still expressing the targets, as well as of the KO populations. For the silencing conditions, the relative expression percentage was derived as follows: first we subtracted the median fluorescence intensity (MFI) of the unstained control and then we normalized the MFI of the transduced M5CAR+ T cells to the MFI of the negative cells within each sample. Relative expression levels were then obtained by normalizing to the scrambled control, where present, or to control M5CART cells. Silencing efficiency was finally calculated as 100% minus the relative expression percentage.

### *In vivo* mouse experiments

The University of Pennsylvania Institutional Animal Care and Use Committee approved all animal experiments, and all animal procedures were performed in the animal facility at the University of Pennsylvania in accordance with Federal and Institutional Animal Care and Use Committee requirements. Female 6–8-week-old NOD-scid IL2Rg^null^ (NSG) mice were purchased from Jackson laboratories and maintained in pathogen free conditions. All experimental protocols were approved by the Institutional Animal Care and Use Committee at the University of Pennsylvania. Mice were maintained in dedicated BSL-2 animal barrier spaces. Animals were injected subcutaneously (sc) with 2 x 10^6^ AsPC-1 CBG GFP cells in 50µl sterile PBS and 50µl Corning matrigel (Catalog# 356234). Once an average tumor volume of 150–200 mm^3^ was reached, 1 x 10^6^ M5CAR+ T cells were injected via tail vein in 200µl sterile PBS. The following T cell groups were tested, each matched for total T-cell dose injected by NTD normalization: NTD, M5CAR T cells, S CD3ζ&β2M M5CAR T cells and KO CD3ζ&β2M M5CAR T cells. No TCRαβ-depleted M5CAR T cells were used. We considered five different T cell donors in two independent experiments. In the first experiment, we employed ND587 and ND610 donors (n= 8–9 mice/group each donor), while in the second experiment, ND584, ND569, and ND627 were used (n= 6–7 mice/group each donor). Mice were evaluated by bi-weekly or weekly tumor volume, bioluminescent imaging (BLI) (Xenogen IVIS-200 spectrum camera), and weight measurements. Tumor volumes were calculated using the formula: volume (mm^3^) = (length x width^2^)/2. BLI data were analyzed using the Living Image software version 4.4 (Caliper LifeSciences, PerkinElmer). Animals were monitored for signs of clinical GVHD as evidenced by >10% loss in body weight, hunching, reduced activity, loss of fur, diarrhea, and conjunctivitis. Mice were euthanized if any tumor dimension exceeded 20 mm, if ulceration covered more than 90% of the tumor surface, if body weight loss was greater than 20%, in cases of disease-related hind limb paralysis, or for severe GVHD, following the approved protocol. A fixed endpoint of 100 days and 66 days after CAR T cell injection to terminate the first and second *in vivo* models, respectively, was defined for remaining mice. Mice that reached these endpoints were euthanized, and necropsy was performed to collect the primary tumor, assess treatment related toxicities and the presence of metastases. Peripheral blood was obtained by retro-orbital bleeding on days 16 (first and second experiment) and 26 (second experiment only) after CAR T cell injection and cell stained for M5CAR and human CD45, CD4, CD8, CD3ϵ, TCRαβ, and β2M markers. At the fixed final endpoint of 66 days after CAR T cell injection (for the second experiment only), three spleens from each donor were collected from the control M5CAR T cell group, nine spleens total, while 4 spleens from each donor were collected from both the S CD3ζ&β2M M5CAR T cell group and the KO CD3ζ&β2M M5CAR T cell group, twelve spleens total for each. Spleens were isolated, dissociated and cell stained for cell viability, M5CAR and for the previous markers. Cell subsets were quantified by flow cytometry using CountBright™ Absolute Counting Beads (BD). All experiments were performed in a randomized fashion.

### Statistical analysis

Survival analysis was performed using the Kaplan Meier estimator and pairwise comparisons using the Log-Rank test. For pairwise comparisons, Wilcoxon Mann-Whitney test or Kruskall-Wallis test followed by Dunn’s *post-hoc* test were used. If the assumptions for parametric tests were met, Student’s T-test or linear regression models were employed. For longitudinal comparisons, linear mixed-effects models with a Gaussian distribution and an identity link were estimated. The model includes independent variables arm, time, and their interaction as fixed effects while subject-specific intercept and slope terms were included as random effects. All p-values were adjusted using the Bonferroni correction to account for multiple arms comparisons. Adjusted p-values were considered significant when below the alpha level 0.05.

For assays conducted on a limited number of T cell donors, non-parametric tests were preferred due to small sample sizes and the likely violation of parametric assumptions. However, given their limited statistical power, these tests could not reliably detect differences or establish statistical significance when biological replicates were limited (e.g., fewer than five in many datasets of this study). In such cases, descriptive statistics, including the mean and standard deviation (SD) or standard error of the mean (SEM), were reported in the graphs. The figure legends explicitly acknowledge the limitations of small sample sizes in accurately representing population variability and the associated challenges in achieving statistical significance.

Statistical analyses were carried out using R Statistical software (The R Foundation for Statistical Computing) version 4.2.3 and GraphPad Prism Version 10.1.1 (270) for macOS. Graphs were created by GraphPad Prism Version 10.1.1 (270) for macOS, BioRender (https://biorender.com) and Adobe Illustrator 2023, all under paid license.

## Results

### Tunable gene silencing or knockout of CD3ζ and β2M genes in T cells

**Figure 1:**
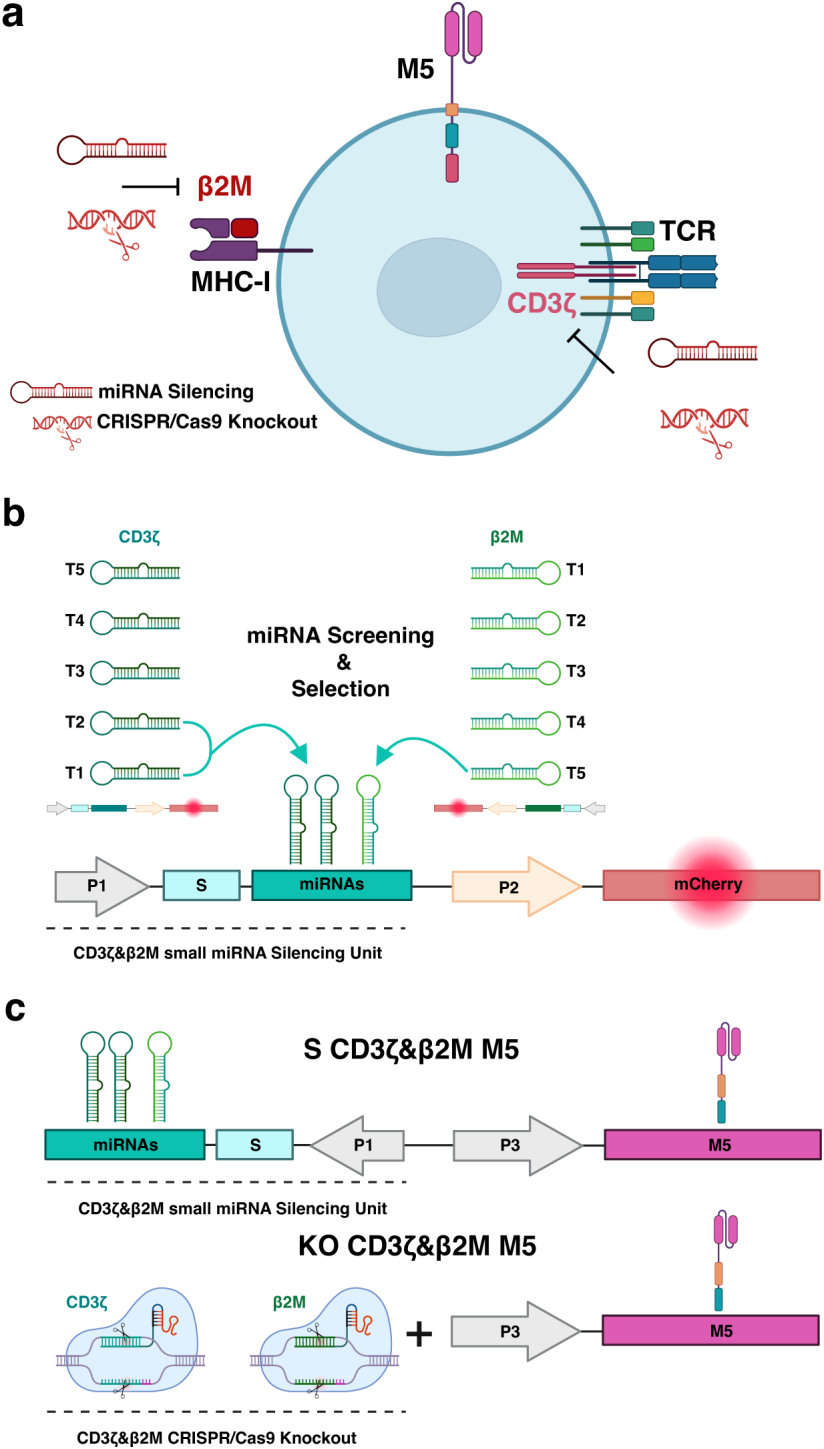
Multiplex genetic engineering employing miRNA-mediated gene silencing as an alternative to CRISPR/Cas9 genome editing in mesothelin-directed CAR (M5CAR) T cells. **(a)**, The effects of miRNA-mediated silencing versus CRISPR/Cas9-based knockout on multiple targets (CD3ζ and β2M) were compared in T cells expressing the M5CAR (M5). The second generation M5CAR is composed of a human Msln-binding scFv (pink) and CD8a hinge and transmembrane (orange) domains fused to 4-1BB (blue) and CD3ζ (red) cytoplasmic signaling domains. **(b)** Screening and selection of the most effective silencing constructs. Five novel target sequences were designed for both CD3ζ and β2M (**Supplementary Table 2**). Each sequence was individually cloned into optimized gene silencing constructs, delivered to T cells via lentiviral transduction, and assessed for silencing efficiency by flow cytometry, using mCherry expression as a reporter for transduced cells. CD3ζ_T1, CD3ζ_T2 and β2M_T5 miRNAs were selected and incorporated into the same silencing construct for CAR T cell engineering. This strategy aimed to achieve complete silencing of the TCR complex and a tuned silencing to retain low surface levels of the MHC-I complex. **(c)** The miRNA expression construct for silencing of CD3ζ and β2M was cloned into the vector coding for the M5CAR in a bidirectional promoter configuration, resulting in dual CD3ζ and β2M silenced (S) M5CAR T cells (S CD3ζ&β2M M5). This design optimized both miRNAs and CAR expression. CRISPR/Cas9 editing employed selected sgRNAs to engineer dual CD3ζ and β2M knockout (KO) M5CAR T cells (KO CD3ζ&β2M M5). Both single and dual CD3ζ and β2M S or KO M5CAR T cells were produced. P1: elongation factor 1 short (EFs) promoter; S: spacer; P2 and P3: human phosphoglycerate kinase (hPGK) promoter.

To evaluate the effects of microRNA (miRNA)-based silencing versus CRISPR/Cas9-mediated deletion on the yield and functional performance of chimeric antigen receptor (CAR) T cells, we conducted proof-of-concept studies targeting the T-cell receptor (TCR) and major histocompatibility complex class I (MHC-I) complexes. These investigations aimed to develop non-alloreactive and hypoimmunogenic CAR T cells (**Figure 1**). We selected miRNAs and single guide RNAs (sgRNAs) that provide comparable repression of TCR expression and differential modulation of MHC-I for our experiments.

### Design and screening of miRNA constructs for silencing TCR and MHC-I

Utilizing methodologies described in Myburgh et al. (12) and online resources for target sequence design, we synthesized miRNAs within a mirGE backbone to target the transcripts of principal subunits of these protein complexes, specifically the CD3ζ and β2-microglobulin (β2M) genes. For CD3ζ, we designed miRNAs targeting mRNA regions outside the coding domain of CD3ζ in the CAR to avoid unintended effects on CAR expression. We considered five target sequences each for CD3ζ and β2M, cloning them individually into optimized gene silencing constructs (**Figure 1b**, **Supplementary Table 2**) (14), and delivering them to T cells via lentiviral transduction. Silencing efficiency was assessed in T cells by flow cytometry, using mCherry expression as a reporter for transduced cells.

**Figure 2:**
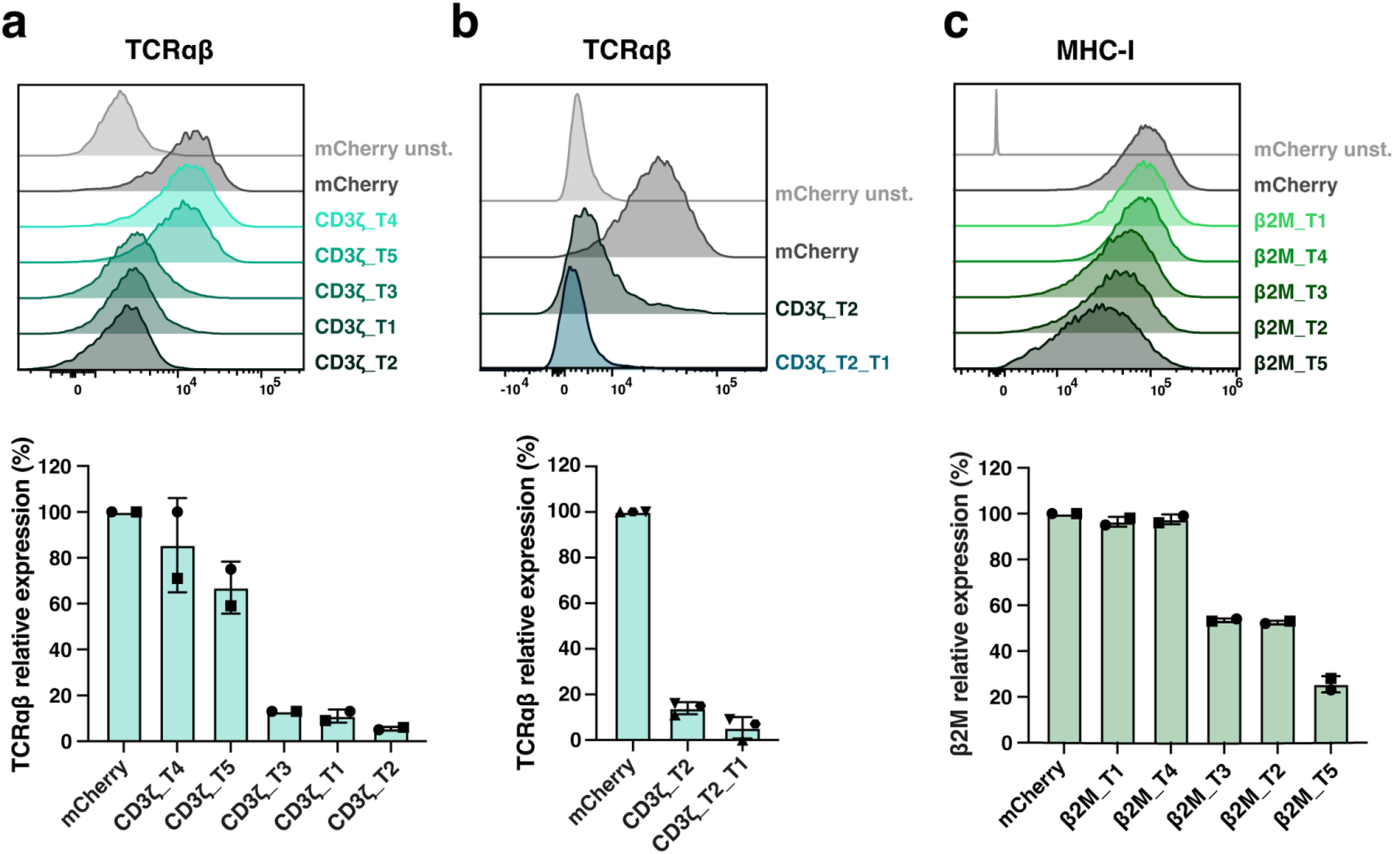
Screening of miRNA target sequences for silencing of TCR and MHC-I expression in primary T cells. **(a–c)**, Five miRNA target sequences (T1 to T5) against transcripts of CD3ζ **(a, b)** or β2M **(c)** were screened, and silencing efficiency was evaluated on transduced mCherry-expressing T cells. CD3ζ_T1, CD3ζ_T2 and β2M_T5 miRNAs were selected. In **(b)**, CD3ζ_T1 and CD3ζ_T2 miRNAs selected in **(a)** were combined (CD3ζ_T2_T1) for TCR optimal silencing. Representative histograms reporting TCR (via TCRαβ) **(a, b)**, and MHC-I (via MHC-I) **(c)** expression levels and their relative expression percentage are shown. N= 2 **(a, c)**, 3 **(b)** T cell donors/condition. Each symbol represents a different T cell donor. Non-parametric tests were used for statistical analysis, but the small sample size prevented statistical significance-driven conclusions. Error bars represent mean (SD).

Three constructs, CD3ζ_T1, CD3ζ_T2, and CD3ζ_T3, demonstrated highly efficient downregulation of TCRαβ expression (**Figure 2a**). Among these, CD3ζ_T2 achieved over 95% silencing of TCR expression, while CD3ζ_T4 and T5 were less effective. To achieve near-complete silencing of the TCR complex and facilitate purification of TCR-deficient cells, we combined the CD3ζ_T1 and CD3ζ_T2 miRNA coding sequences in the final expression construct (**Figures 1b**, **2b**).

Regarding MHC-I, the β2M_T2 and T3 constructs resulted in mean (SD) silencing efficiencies of 47.5% (0.7) and 46.5% (0.7), respectively, while β2M_T1 and T4 had no apparent effect (**Figure 2c**). The β2M_T5 construct was the most efficient, achieving a 74.5% (3.5) reduction in MHC-I expression in T cells. To potentially protect modified T cells from a natural killer (NK) cell ‘missing self’ response, deeper silencing of β2M was not pursued. Consequently, the CD3ζ_T1, CD3ζ_T2, and β2M_T5 miRNA coding sequences were chosen for CAR T-cell engineering (**Figure 1b**) and cloned into the vector coding for the M5CAR in a bidirectional promoter configuration, resulting in dual CD3ζ and β2M silenced (S) M5CAR T cells (S CD3ζ&β2M M5CAR T cells) (**Figure 1c**).

### CRISPR/Cas9 knockout of TCR and MHC-I

For CRISPR/Cas9 knockout (KO) of MHC-I and TCR complexes, we utilized a sgRNA targeting the β2M gene, as designed for NCT05037669 (**Supplementary Table 1**). We also designed and screened multiple sgRNAs targeting regions outside the CD3ζ domain coding region in the CAR on T cells (**Supplementary Table 1**). Among them, sgRNA_4 was selected for its high KO efficiency, resulting in only 2.1% (0.4) CD3ϵ+ T cells with the lowest median fluorescence intensity (MFI) (**Supplementary Figures 1A–D**). Simultaneous deletions of CD3ζ and β2M in CAR-modified T cells targeting CD19 were efficient and comparable to single-target deletions (**Supplementary Figures 1E–I**). The selected sgRNAs were subsequently used to engineer dual CD3ζ and β2M KO M5CAR T cells (KO CD3ζ&β2M M5CAR T cells) and compared to miRNA-mediated silencing (**Figure 1c**).

### Superior expansion, preservation of cell viability, and memory phenotypes in gene-silenced compared to CRISPR/Cas9-edited M5CAR T cells

The miRNA and M5CAR cassettes were expressed from the same lentiviral vector. Both single and dual CD3ζ and β2M S M5CAR T cells, including a scrambled control, were generated using the same protocol as control M5CAR T cells (**Supplementary Figure 2A**). In contrast, single and dual CD3ζ and β2M KO M5CAR T cells, along with control Mock M5CAR T cells, were produced using a protocol that included a CRISPR/Cas9 NF step prior to activation and transduction (**Supplementary Figure 2B**), as previously reported (18).

**Figure 3:**
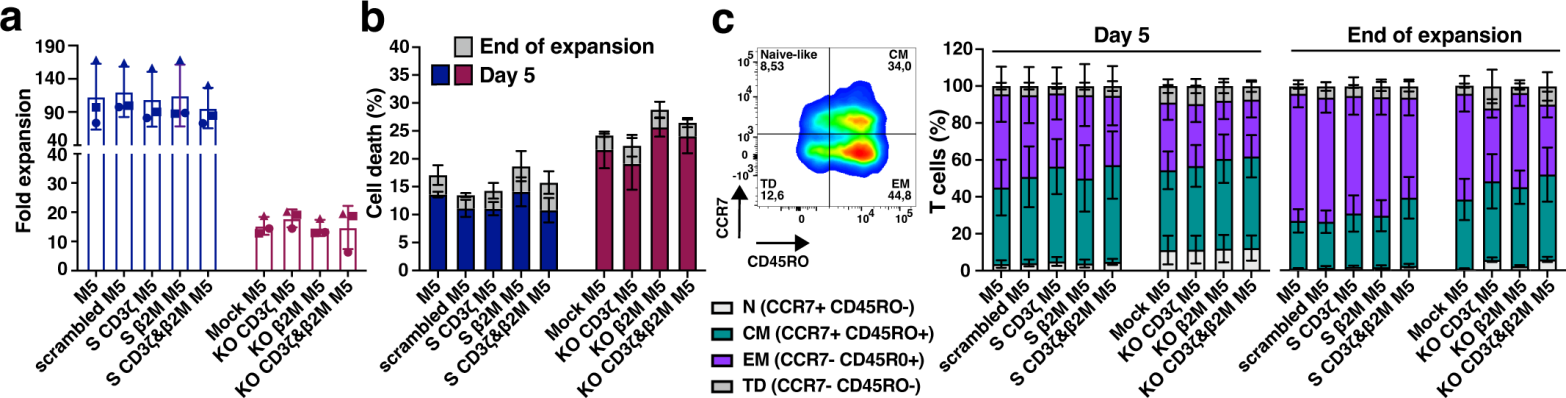
Enhanced expansion, viability, and better-preserved memory phenotypes in silenced M5CAR T cells versus CRISPR/Cas9-edited ones. **(a)**, Fold expansion of M5CAR T cells expressed as the ratio of the final number of cells to the initial number of cells. For KO M5CAR T cell groups, the cell number after nucleofection was used as the initial value. M5CAR T cells, M5; M5CAR T cells with scrambled miRNA (scrambled M5); M5CAR T cells with miRNAs for single silenced (S) CD3ζ (S CD3ζ M5) or β2M (S β2M M5) transcripts; M5CAR T cells with miRNAs for combined silencing of CD3ζ and β2M (S CD3ζ&β2M M5); Mock-edited M5CAR T cells (Mock M5); M5CAR T cells with sgRNAs for single knockout (KO) of CD3ζ (KO CD3ζ M5) or β2M (KO β2M M5) genes; M5CAR T cells with combined sgRNAs for dual KO of CD3ζ and β2M genes (KO CD3ζ&β2M M5CAR). **(b)**, Cell death of M5CAR T cells on day 5 and at the end of expansion, measured using the LIVE/DEAD™ Fixable Dead Cell Stain Kit. **(c)**, T cell memory subsets in M5CAR+ T cells by CD45RO and CCR7 markers on day 5 and at the end of expansion. Naïve-like, N; Central memory, CM; effector memory, EM; terminally differentiated, TD. N= 3 T cell donors/group. In **(a)**, each symbol represents a different T cell donor (◼ ND587; ▲; ND610; ● ND561). Non-parametric tests were used for statistical analysis, but the small sample size prevented statistical significance-driven conclusions. Error bars represent mean (SD) **(a)** and mean (SEM) **(b, c)**.

S M5CAR T cells, including S CD3ζ&β2M M5CAR T cells, exhibited expansion rates and cell size changes comparable to those of control M5CAR T cells (**Supplementary Figures 3A, B**). In contrast, the expansion of KO M5CAR T cells was negatively impacted by gene editing via NF (**Supplementary Figures 3C, D**). In all KO groups, including the KO CD3ζ&β2M M5CAR T cell group, NF on day 0 resulted in 40-50% reduction in cell numbers. Moreover, their expansion was impaired. Using the post-NF cell count as the baseline, KO cells showed significantly lower population doublings (PDs). This impairment was particularly evident before debeading on day 5, as KO cells displayed a longer and more pronounced lag phase compared to S cells. After day 5, KO and S cells expanded at comparable rates; however, KO cells failed to reach the same overall levels due to the initial deficit. The mean (SD) fold expansion of S CD3ζ&β2M M5CAR T cells from three and five T cell donors was 96 (30.4) (**Figure 3a**) and 90.5 (24.9) (**Supplementary Figure 3E**), respectively, comparable to those of single-S and control M5CAR T cell groups. The KO CD3ζ&β2M M5CAR T cell group showed markedly lower fold expansions of 14.8 (7.4) (**Figure 3a**) and 13.9 (5.4) (**Supplementary Figure 3E**), similar to the other KO groups. Together, these results indicate an approximately six fold difference in expansion yield between miRNA- and KO-modified M5CAR T cells. Starting with a higher number of cells for manufacturing in the KO groups, as specified in Materials and Methods and **Supplementary Figure 2**, compensated for these differences in expansion, resulting in a total number of cells after expansion comparable to that of the S groups.

The viability of S CD3ζ&β2M M5CAR T cells after bead removal on day 5 and at the end of expansion was similar to that of control M5CAR T cells (**Figure 3b**, **Supplementary Figure 3F**). In contrast, the viability of KO CD3ζ&β2M M5CAR T cells, as well as that of the single KO groups, was reduced due to NF and gene editing, leading to significant cell loss and a slightly higher rate of cell death compared to non-nucleofected groups (**Figure 3b**, **Supplementary Figure 3F**). The proportions of CD4+ and CD8+ cells were largely comparable across the M5CAR+ control, dual S, and dual KO groups, although overall CD4+ and CD8+ proportions varied noticeably between T cell donors, irrespective of the CAR T cell groups (**Supplementary Table 3**).

Finally, S CD3ζ&β2M M5CAR T cells exhibited a memory profile similar to that of control M5CAR T cells, with only a slightly higher percentage of central memory (CM) T cells and a lower percentage of effector memory (EM) T cells at both day 5 and the end of expansion (**Figure 3c**, **Supplementary Figure 3G**). In contrast, KO CD3ζ&β2M M5CAR T cells displayed a slightly higher percentage of naïve-like T cells on day 5 and throughout expansion, along with a marginally increased proportion of CM and terminally differentiated (TD) T cells and a decreased percentage of EM T cells compared to control and S CD3ζ&β2M M5CAR T cells at both time points (**Figure 3c**, **Supplementary Figure 3G**). This pattern of memory T cell subtypes was also observed in KO CD3ζ M5CAR T cells but not in the other groups. The expression of activation markers (4-1BB, TIGIT, PD-1, TIM-3, LAG-3, and OX40) showed no significant differences between S and KO M5CAR+ T cells and remained comparable to control M5CAR+ T cells (**Supplementary Figure 4**).

Overall, S M5CAR T cells demonstrated expansion and viability comparable to control M5CAR T cells, with similar memory profiles, albeit with a slight shift toward higher CM and lower EM T cell levels. In contrast, KO M5CAR T cells exhibited reduced expansion, lower viability, and less favorable memory phenotypes, retaining lower percentages of naïve-like T cells while showing an increased proportion of TD and CM cells and a reduced proportion of EM cells by the end of expansion. These findings indicate that miRNA-based gene silencing preserves the production yield of phenotypically desirable CAR T cells, unlike CRISPR/Cas9 KO via NF.

### Multiplex gene silencing and simultaneous CAR expression in S CD3ζ&β2M M5CAR T cells

**Figure 4:**
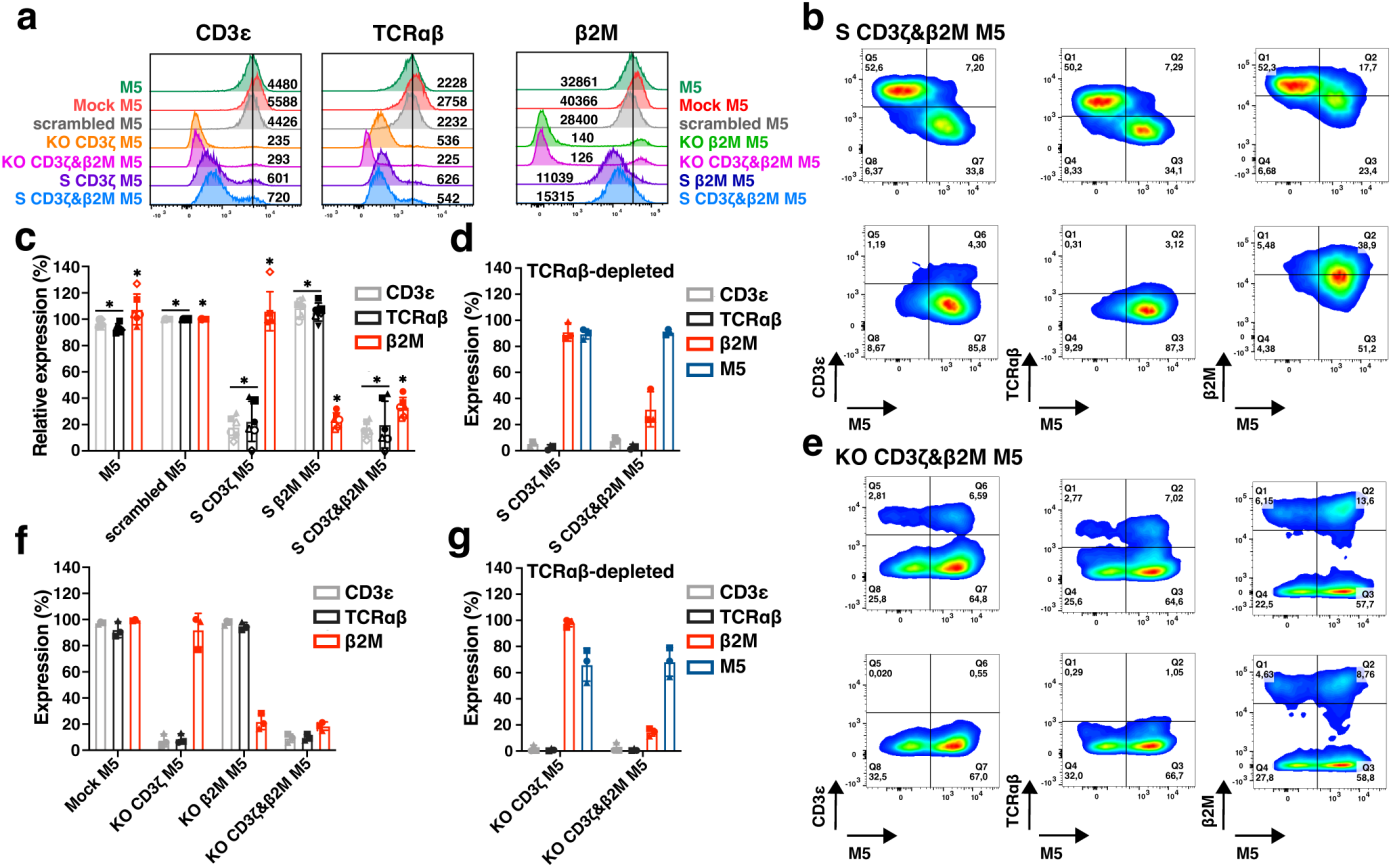
Comparison of multiplex silencing versus deletion of TCR and MHC-I complexes in M5CAR T cells before and after TCRαβ depletion. **(a)**, Representative histograms, and median fluorescence intensity, reporting TCR (via CD3ϵ and TCRαβ) and MHC-I (via β2M) expression levels in silenced (S) and knockout (KO) M5CAR+ T cells. **(b)**, Representative dot plots of dual CD3ζ and β2M S M5CAR T cells (S CD3ζ&β2M M5) before (top row) and after (bottom row) TCRαβ depletion. M5CAR (M5) expression is shown on the x-axis, with TCR (via CD3ϵ and TCRαβ) and MHC-I (via β2M) complexes on the y-axis. **(c)**, Relative expression percentages of TCR and MHC complexes in S M5CAR T cells and in M5CAR T cells at the end of expansion, normalized to scramble M5CAR T cells. In S CD3ζ&β2M M5CAR T cells, mean (SD) percentages were 13.1% (3.6) for CD3ϵ, 8.9% (6.7) for TCRαβ, and 32.3% (9.3) for β2M, which result to surface silencing efficiencies of 86.9% (3.6) and 91.1% (6.7) for TCR via CD3ϵ and TCRαβ, respectively, and 67.7% (9.3) for MHC-I via β2M. **(d)**, Expression of TCR (via CD3ϵ and TCRαβ) and MHC-I (via β2M) complexes and M5CAR (M5) after TCRαβ depletion in M5CAR T cells with either single CD3ζ (S CD3ζ M5) or dual CD3ζ and β2M (S CD3ζ&β2M M5) silencing. **(e)**, Representative dot plots of dual CD3ζ and β2M KO M5CAR T cells (KO CD3ζ&β2M M5) before (top row) and after (bottom row) TCRαβ depletion. **(f)**, Expression of TCR and MHC complexes on KO M5CAR T cells at the end of expansion. Low percentages of KO CD3ζ&β2M M5CAR T cells still expressed CD3ϵ, TCRαβ, and β2M, corresponding to mean (SD) KO efficiencies of 90.9 (3.2) %, 90.3 (2.4) %, and 81.6 (3.2) % for TCR via CD3ϵ and TCRαβ, and for MHC-I, respectively. **(g)**, Expression of TCR (via CD3ϵ and TCRαβ) and MHC-I (via β2M) complexes and M5CAR (M5) after TCRαβ depletion in M5CAR T cells with either single CD3ζ (KO CD3ζ M5) or dual CD3ζ and β2M (KO CD3ζ&β2M M5) gene deletion. N= 4-6 **(c)**, 3 **(d, f, g)** T cell donors/group. Each symbol represents a different T cell donor (◼ ND587; ▲; ND610; ● ND561; ◇ ND541; ○ ND224; △ ND582). P value (*p ≤ 0.05) was determined by Wilcoxon Mann-Whitney test. In **(d, f, g)**, non-parametric tests were used for statistical analysis, but the small sample size prevented statistical significance-driven conclusions. Error bars represent mean (SD).

Gene silencing and KO efficiency were assessed by evaluating TCR expression (via CD3ϵ and TCRαβ) and MHC-I expression (via β2M) (**Figure 4a**). The mean fluorescence intensities (MFIs) of CD3ϵ and TCRαβ in S CD3ζ&β2M M5CAR T cells were slightly higher than those observed in KO CD3ζ&β2M M5CAR T cells but remained lower than in control and scrambled M5CAR T cells. Silencing of β2M resulted in reduced β2M expression compared to control and scrambled M5CAR T cells, whereas β2M was almost entirely absent on the surface of KO CD3ζ&β2M M5CAR T cells (**Figure 4a**).

CD3ζ and β2M silencing in M5CAR T cells was evaluated before TCRαβ depletion, confirming that M5CAR+ transduced cells exhibited lower expression levels of both TCR and MHC-I compared to their non-transduced M5CAR- counterparts (**Figure 4b**, top row). Following TCRαβ depletion, the selected transduced M5CAR+ cells retained miRNA-mediated silencing (**Figure 4b**, bottom row). In S CD3ζ&β2M M5CAR T cells, gene silencing was efficient, achieving reductions in surface expression of 86.9% (3.6) and 91.1% (6.7) for TCR via CD3ϵ and TCRαβ, respectively, and 67.7% (9.3) for MHC-I via β2M (**Figure 4c**). These results were comparable to those of single-S CD3ζ or β2M M5CAR T cells. After TCRαβ depletion, S CD3ζ&β2M M5CAR T cells were nearly 100% M5CAR+ and lacked detectable TCR expression (**Figure 4D**). Additionally, MHC-I silencing was retained, with residual β2M expression at 31.7% (13.5) of control M5CAR T cell levels (**Figure 4d**).

Production of KO CD3ζ&β2M M5CAR T cells resulted in the KO of target genes in both CAR-transduced and non-transduced T cells (**Figure 4e**, top row). TCRαβ depletion yielded a mixed population of M5CAR+ T cells and non-transduced T cells, both carrying the KO, with a subset still expressing β2M (**Figure 4e**, bottom row). Prior to TCRαβ depletion, a low percentage of KO CD3ζ&β2M M5CAR T cells retained expression of CD3ϵ, TCRαβ, and β2M (**Figure 4f**). After TCRαβ depletion, KO CD3ζ&β2M M5CAR T cells maintained the same percentage of β2M+ and M5CAR+ cells as observed at the end of manufacturing (**Figure 4g**).

Analysis of additional T cell donors confirmed the low relative expression of CD3ϵ, TCRαβ, and β2M in S CD3ζ&β2M M5CAR T cells (**Supplementary Figure 5A**), as well as the low frequency of these markers in KO CD3ζ&β2M M5CAR T cells (**Supplementary Figure 5B**). After TCRαβ depletion, the percentage of cells still expressing CD3ϵ, TCRαβ, and β2M at levels comparable to control M5CAR T cells was similar between S CD3ζ&β2M and KO CD3ζ&β2M M5CAR T cells. However, only in TCRαβ-depleted S CD3ζ&β2M M5CAR T cells was a higher percentage of M5CAR+ cells achieved (**Supplementary Figure 5C**). Among the T cell donors analyzed in **Supplementary Figure 5C**, the mean (SD) yield of TCRαβ depletion was slightly lower for S CD3ζ&β2M M5CAR T cells than for KO CD3ζ&β2M M5CAR T cells, though the difference was not statistically significant.

At the end of T cell expansion, all gene-S groups had a lower percentage of transduced T cells expressing M5CAR compared to control M5CAR T cells and KO groups (**Supplementary Figures 5D, E**). Additionally, CAR MFIs in S M5CAR T cells appeared lower than in control M5CAR T cells, whereas some KO donors exhibited higher CAR MFIs, although the difference was not statistically significant (**Supplementary Figures 5F, G**). The reduced M5CAR+ T cell levels and M5CAR MFIs in S groups likely stem from lower gene transfer efficiency and/or expression levels inherent to the multiplexed constructs, suggesting that further optimization of miRNA-carrying vectors and experimental conditions may be required.

**Figure 5:**
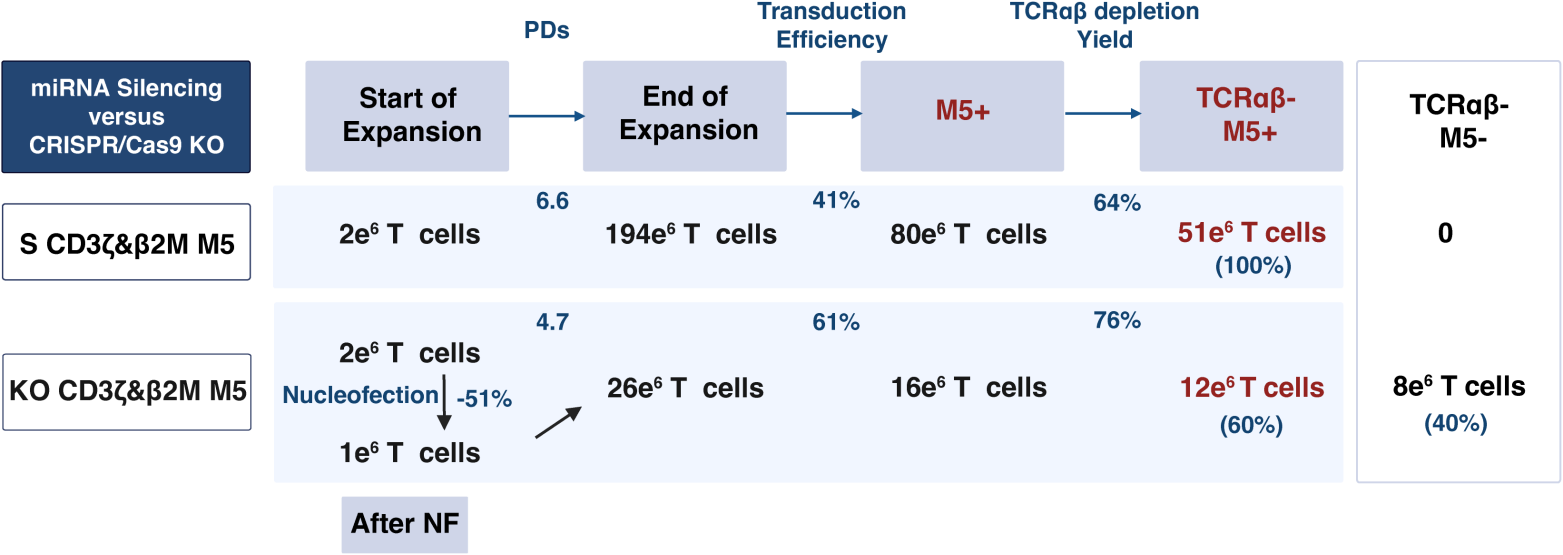
MiRNA-mediated silencing achieves a higher yield and purer population of non-alloreactive and hypoimmunogenic M5CAR T cells compared to CRISPR/Cas9-mediated editing. This table illustrates the yield and characteristics of the final product when starting with an equal number of T cells (e.g., 2 x 10^6^ cells) for both silenced (S) CD3ζ&β2M M5CAR T cells (S CD3ζ&β2M M5) and knockout (KO) CD3ζ&β2M M5CAR T cells (KO CD3ζ&β2M M5). Factors considered include cell loss after nucleofection (NF) in KO CD3ζ&β2M M5CAR T cells, population doublings (PDs), transduction efficiency, and yield of TCRαβ depletion, with values based on the average from five unique T cell donors. The derived cell numbers are approximations based on these factors and rounding. For S CD3ζ&β2M M5CAR T cells, the final product would likely be a pure population of TCRαβ-depleted CAR+ T cells with MHC-I silencing. In contrast, for KO CD3ζ&β2M M5CAR T cells, the final product would consist of TCRαβ-depleted cells, including both CAR+ and non-transduced T cells, all carrying the KO. A subset of these cells would still express β2M, with the proportions of CAR+ T cells and β2M+ T cells determined by the efficiencies of transduction and KO, respectively.

Several processing factors influence the final product, including cell loss after NF in ko M5CAR T cells, PDs, transduction efficiency, and TCRαβ depletion yield. Assuming an equal initial manufacturing count of T cells (e.g., 2 × 10^6^ cells) for both S CD3ζ&β2M and KO CD3ζ&β2M M5CAR T cells, distinct differences in the yield and composition of the final product can be illustrated using the average values of these factors from five unique T cell donors (**Figure 5**). Based on the donors and methods employed in this study, the fold expansion of M5CAR+ T cells is estimated to be approximately four times higher in S compared to KO M5CAR T cells. The final product for S CD3ζ&β2M M5CAR T cells consists of a highly pure population of TCRαβ-depleted M5CAR+ T cells with MHC-I silencing. In contrast, the final product for KO CD3ζ&β2M M5CAR T cells is a heterogeneous mixture of TCRαβ-depleted cells, including both M5CAR+ and M5CAR- T cells with β2M KO, as well as M5CAR+ and M5CAR- T cells still expressing β2M. The proportions of M5CAR+ and β2M+ T cells depend on transduction and KO efficiencies.

Overall, miRNA-mediated gene silencing resulted in significantly higher fold expansion and a nearly pure population of modified M5CAR T cells compared to CRISPR/Cas9-mediated KO.

### S CD3ζ&β2M M5CAR T cells retain significant tumor-killing capacity *in vitro*

**Figure 6:**
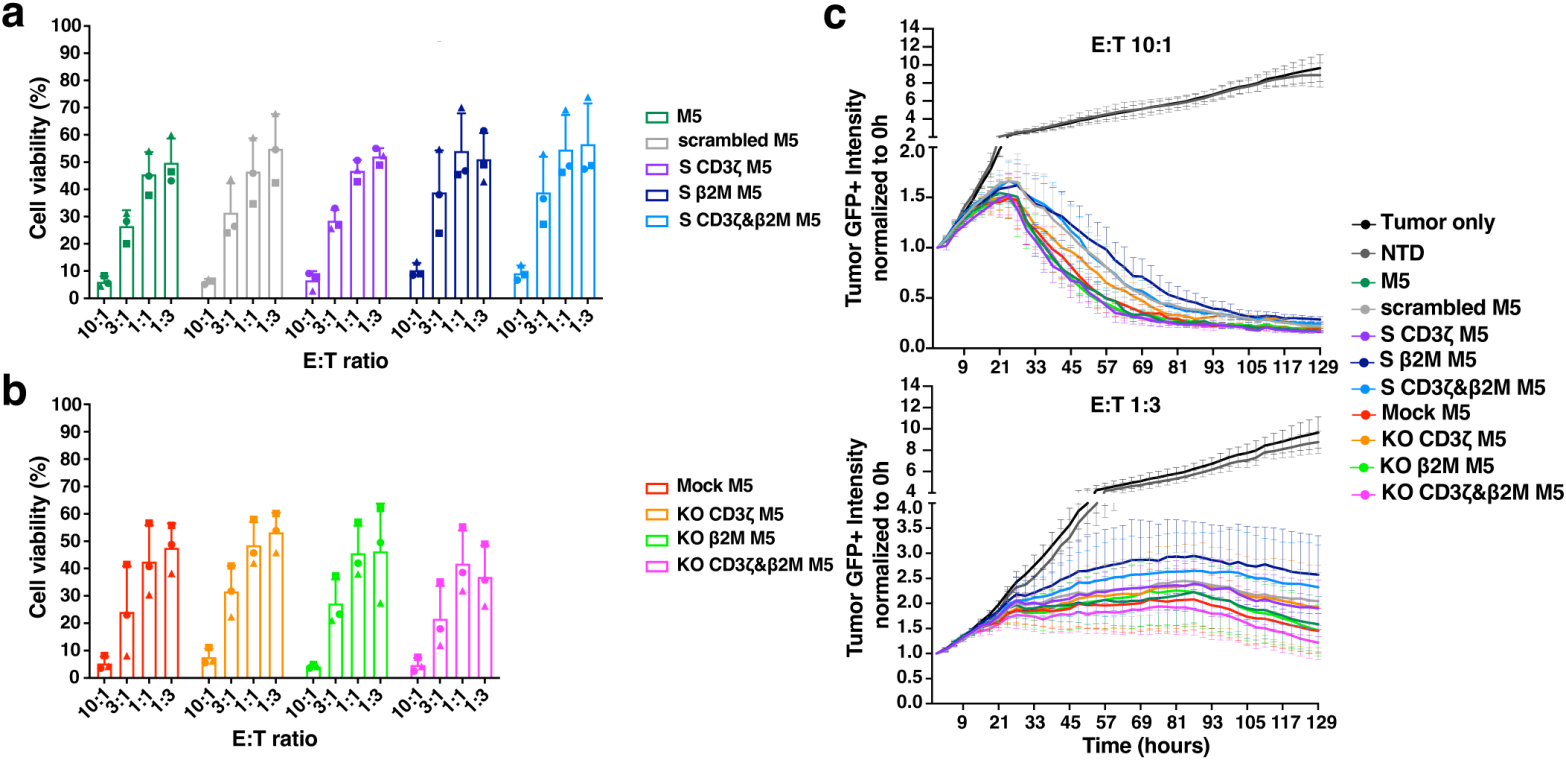
Efficient *in vitro* cytotoxicity by TCR and MHC-I dual silenced (S CD3ζ&β2M) M5CAR T cells. **(a–c)**, Viability of AsPC-1 tumor cells after co-culture with silenced (S) and knockout (KO) M5CAR T cells measured by bioluminescence imaging at a 48-hour endpoint **(A, B)** and in real-time over more than 5 days of co-culture **(C)**. N= 3 T cell donors/group. Each symbol represents a different T cell donor (◼ ND587; ▲; ND610; ● ND561). Non-parametric tests were used for statistical analysis, but the small sample size prevented statistical significance-driven conclusions. Error bars represent mean (SD).

The cytotoxicity of miRNA- and CRISPR-modified CAR T cells was assessed *in vitro* by co-culturing with the human PDAC Msln+ cell line AsPC-1. Both single and dual CD3ζ and β2M S M5CAR T cells exhibited potent tumor-killing ability, with no significant differences observed between groups in endpoint assays (**Figures 6a, b**). In real-time co-culture, S M5CAR T cells effectively eliminated tumor cells, albeit at a slightly slower rate compared to KO or control M5CAR T cells (**Figure 6c**, **Supplementary Figure 6A**). After six days of co-culture, cells were collected and analyzed by flow cytometry to assess potential differences among groups across T cell donors. The memory phenotype of M5CAR+ T cells was similar across all groups, with the majority exhibiting an effector memory (EM) profile (**Supplementary Figure 6B**). Gene silencing and KO of target genes were confirmed at the end of the co-culture (**Supplementary Figure 6C**).

Expansion rates for M5CAR+ and M5CAR- T cells, M5CAR MFI (normalized to the control group), and the percentage of M5CAR+ T cells post-co-culture were also evaluated (**Supplementary Figures 6D–H**). Despite donor variability, M5CAR+ T cells in both the S CD3ζ&β2M and KO CD3ζ&β2M M5CAR T cell groups generally expanded less than control M5CAR T cells. Some donors also exhibited increased M5CAR- T cell populations in the S and KO groups at various effector-to-target (E:T) ratios (**Supplementary Figures 6D, E**). For the percentage of M5CAR+ T cells over total live cells in co-culture and the M5CAR MFI of the M5CAR+ T cell population, trends remained consistent across all E:T ratios, with the S CD3ζ&β2M M5CAR T cell group showing, on average, lower percentages and MFIs (**Supplementary Figures 6F, G**). Data summarizing treatment group comparisons for each T cell donor are presented in **Supplementary Table 4**. In donors where scrambled, S CD3ζ, and S β2M M5CAR T cell groups were evaluated (ND587, ND561, and ND610), similar trends were observed as in the S CD3ζ&β2M M5CAR T cell condition (**Supplementary Figure 6H**), suggesting that the lower proportions of M5CAR+ T cells were independent of the specific silencing targets.

Overall, these findings indicate a deficiency in the expansion of both S CD3ζ&β2M and KO CD3ζ&β2M M5CAR T cells, with a more pronounced effect on M5CAR+ T cells than on M5CAR- T cells. This discrepancy, noting a higher tendency for CAR downregulation following antigen stimulation, has been reported previously (17). Consistent with observations at the end of T cell expansion, post-co-culture M5CAR MFIs in S CD3ζ&β2M M5CAR T cells remained lower than in control and KO M5CAR T cells, whereas M5CAR MFIs in KO CD3ζ&β2M M5CAR T cells were slightly lower, comparable, or, in some cases, higher than in control M5CAR T cells. Despite variability among donors, differences in expansion, and a slower tumor-killing rate, both endpoint and real-time assays demonstrated that S M5CAR T cells effectively controlled tumor growth.

### Enhanced metastatic control by S CD3ζ&β2M M5CAR T cells compared to KO CD3ζ&β2M M5CAR T cells, with similar antitumor effects on primary tumors

**Figure 7:**
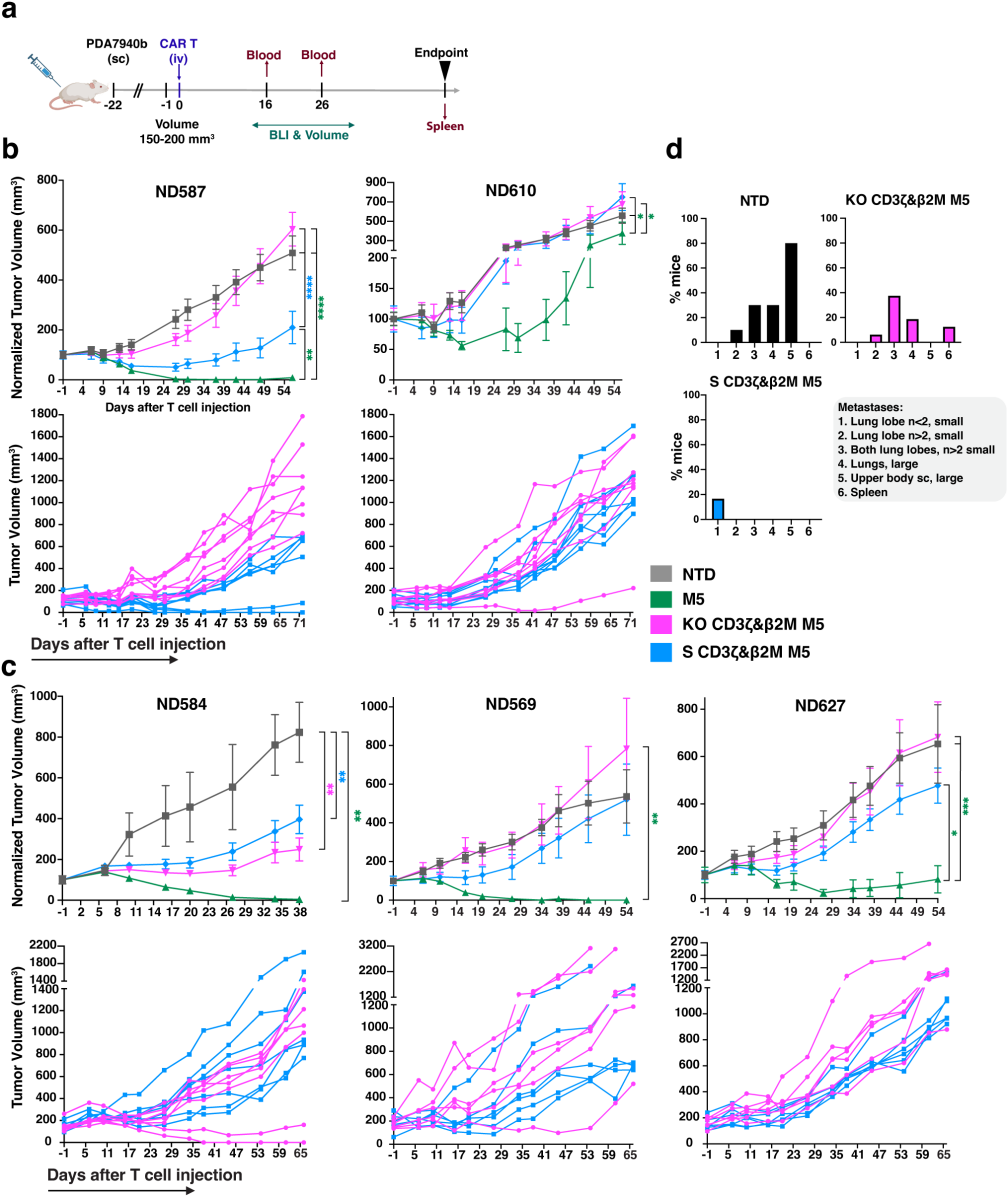
Improved metastatic control of S CD3ζ&β2M M5CAR T cells relative to KO CD3ζ&β2M M5CAR T cells, with similar antitumor effects on primary tumors. **(a)**, Schematic of *in vivo* experiments evaluating the anti-tumor efficacy of silenced (S) and knockout (KO) M5CAR T-cell therapy against PDAC. Animals were injected subcutaneously (sc) with 2 x 10^6^ AsPC-1 CBG GFP cells. Once an average tumor volume of 150–200 mm^3^ was reached, 1 x 10^6^ M5CAR+ T cells were injected via tail vein (iv). The following T cell groups were tested, each matched for total T-cell dose injected by NTD normalization: NTD, M5CAR T cells, S CD3ζ&β2M M5CAR T cells and KO CD3ζ&β2M M5CAR T cells. No TCRαβ-depleted M5CAR T cells were used. Two independent experiments were conducted, with a total of five different T cell donors, the first using ND587 and ND610 (n= 8–9 mice/group each donor), and the second ND584, ND569, and ND627 (n= 6–7 mice/group each donor). These donors were selected based on the characterization of M5CAR T cells after six days of co-culture with tumor cells, aiming to encompass all the variability detected among T cell donors and M5CAR T cell groups. Mice were euthanized per protocol if tumor dimensions exceeded 20 mm, ulceration covered over 90% of the tumor surface, or body weight loss exceeded 20%. A fixed endpoint of 100 days for the first experiment and 66 days for the second experiment after CAR T-cell injection was set for the remaining mice. **(b, c)**, Tumor volume over time, either normalized to baseline volume at day -1 from CAR T cell injection (top rows) or presented as non-normalized individual mouse curves for the S CD3ζ&β2M and KO CD3ζ&β2M M5CAR T cell groups (bottom rows), up to the point when a representative number of mice remained in each group, as indicated on the x-axis. Non-normalized individual mouse curves are shown only for the S CD3ζ&β2M and KO CD3ζ&β2M M5CAR T cell groups to provide a more detailed visualization of tumor progression in the two groups under direct comparison, beyond the time point at which normalized plots including all groups become less informative due to the decreasing number of surviving animals. **(d)**, In the second experiment, necropsy was performed on day 66 after CAR T cell injection. The histograms for each treatment group show the percentage of mice carrying metastases, with characteristics and tissue distribution indicated by the numeric legend (1 to 6) as follows: (1) n<2 small metastases in one lung lobe; (2) n>2 small metastases in one lung lobe; (3) n>2 small lung metastases in both lung lobes; (4) large lung metastases; (5) metastases in the flank of the primary tumor extending toward the upper arm, located subcutaneously outside the peritoneum; (6) spleen metastases. N= 10, 15, 16, and 18 mice for NTD, control M5CAR T cell, KO CD3ζ&β2M M5CAR T cell, and S CD3ζ&β2M M5CAR T cell groups, respectively. P values (*p ≤ 0.05; **p ≤ 0.01; ***p ≤ 0.001; ****p ≤ 0.0001) were calculated by mixed-effects model with a Gaussian distribution and an identity link. Error bars represent mean (SEM).

To compare the antitumor activity of S CD3ζ&β2M and KO CD3ζ&β2M M5CAR T cells, we conducted *in vivo* experiments using a subcutaneous PDAC model in NSG mice (**Figure 7a**). The first study, involving two T cell donors, ran for 100 days post-CAR T cell injection (**Figure 7b**), while the second study, with three donors, lasted 66 days (**Figures 7c, d**). Both S CD3ζ&β2M and KO CD3ζ&β2M M5CAR T cells demonstrated similar efficacy in controlling primary tumor growth, though responses varied between donors (**Figures 7b, c**). In all groups, tumor growth was effectively controlled during the initial weeks. Control M5CAR T cells exhibited the most potent and sustained tumor suppression across all donors, except for ND610 (**Figures 7b, c**). Survival was generally poorest in the NTD group, particularly for donors ND587 and ND584 (**Supplementary Figures 7A, B**). In donor ND569, the lowest survival was observed in the M5CAR T cell group, attributed to graft-versus-host disease (GVHD), suggesting a stronger xenogeneic response. Survival mediated by S CD3ζ&β2M and KO CD3ζ&β2M M5CAR T cells was comparable in ND584, slightly improved with S cells in ND587, ND569, and ND627, and better with KO cells in ND610 (**Supplementary Figures 7A, B**). Symptoms of GVHD, including >10% weight loss, hunching, reduced activity, fur loss, and conjunctivitis, were almost exclusively observed in the control M5CAR T cell group, affecting 13 out of 34 mice (38.2%) across both experiments, particularly in donors ND587 and ND569. GVHD symptoms were detected in only one mouse treated with KO CD3ζ&β2M M5CAR T cells and were completely absent in the S CD3ζ&β2M M5CAR T cell group.

The causes of death for each treatment group and T cell donor are detailed in **Supplementary Tables 6** and **7**, corresponding to the first and second *in vivo* studies, respectively.

At the fixed endpoint of 66 days post-injection in the second study, necropsy and a semiquantitative assessment of metastases were performed (**Figure 7d**, **Supplementary Table 5**). Lung metastases were the most common, appearing as either isolated small masses (fewer than two), small tumors covering the surface of one or both lung lobes, or large masses. Additional metastases were observed in the spleen and in the same flank as the primary tumor, extending toward the upper arm in the subcutaneous tissue outside the peritoneum. S CD3ζ&β2M M5CAR T cells were more effective than KO CD3ζ&β2M M5CAR T cells in preventing metastasis formation. In the S CD3ζ&β2M M5CAR T cell group, small, isolated lung metastases in a single lung lobe were detected in only 3 out of 18 mice (16.7%) (**Figure 7d**). In contrast, mice treated with KO CD3ζ&β2M M5CAR T cells exhibited higher metastatic burden, with 6.25% showing diffuse small lung metastases in one lobe, 37.5% with small metastases in two lobes, 18.8% with large lung metastases, and 12.5% with spleen metastases (**Figure 7d**). No metastases were found in the control M5CAR T cell group, while the non-transduced T cell (NTD) group exhibited significantly higher metastatic rates than all other groups (**Figure 7d**). Overall, the S CD3ζ&β2M M5CAR T cell group had significantly fewer lung metastases than both the NTD and KO CD3ζ&β2M M5CAR T cell groups.

These findings demonstrate that S CD3ζ&β2M M5CAR T cells were as effective as KO CD3ζ&β2M M5CAR T cells in controlling primary tumor growth. However, S CD3ζ&β2M M5CAR T cells provided superior metastatic control, highlighting the potential advantage of gene silencing over CRISPR-mediated KO in preventing tumor spread.

### S CD3ζ&β2M M5CAR T cells exhibit greater persistence and stable target gene silencing *in vivo*

**Figure 8:**
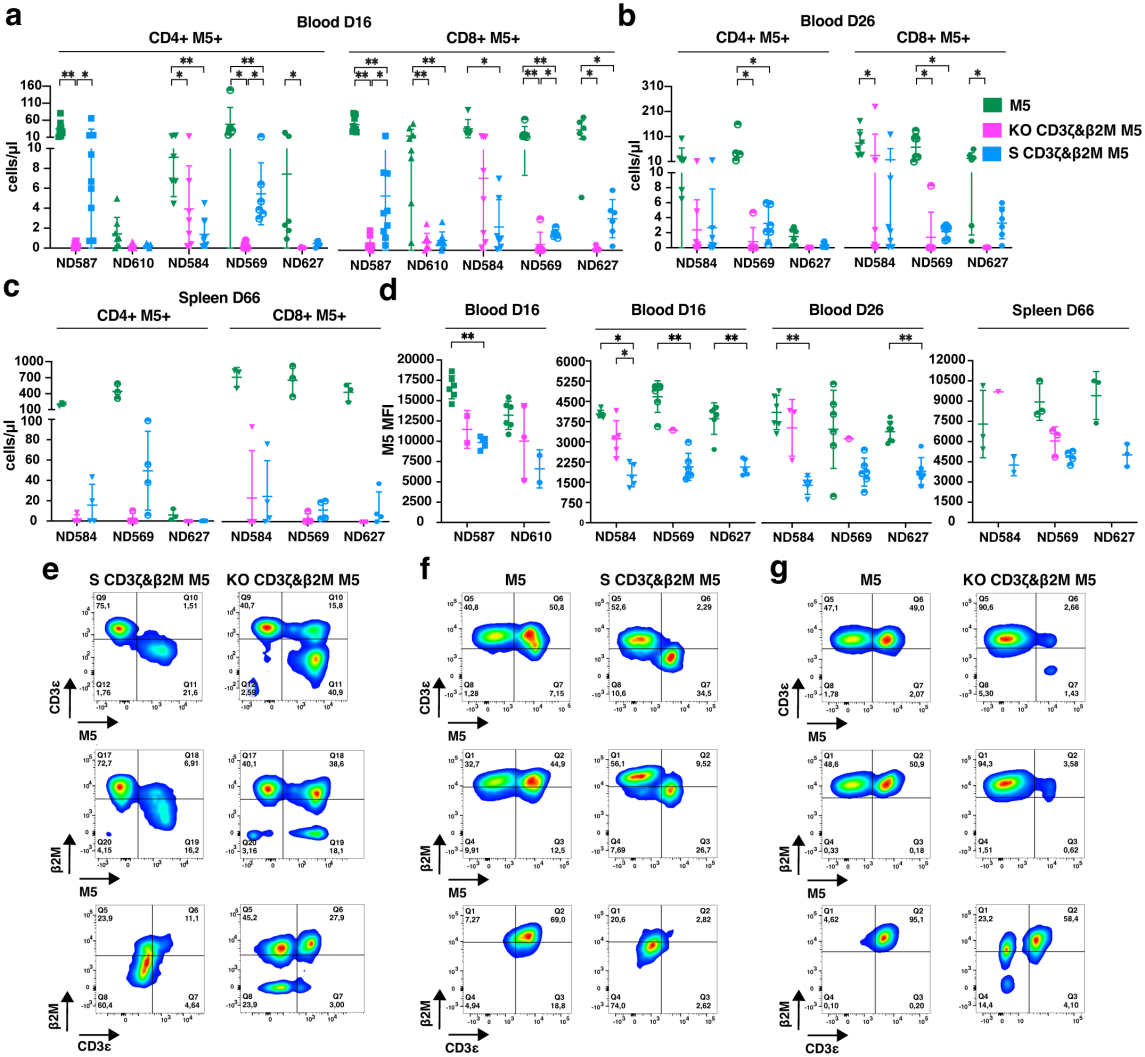
Greater persistence of S CD3ζ&β2M M5CAR T cells than KO CD3ζ&β2M M5CAR T cells and stable target gene silencing *in vivo*. **(a–c)**, Concentration of CD4+ and CD8+ silenced (S) and knockout (KO) M5CAR (M5)+ T cells in mouse blood on days 16 **(a)** and 26 **(b)**, and in the spleen on day 66 **(c)** after injection, for all specified donors. **(d)**, M5CAR MFI of M5CAR T cells detected in the blood at days 16 and 26, and in the spleen on day 66 after injection. For the blood, n= 8–9 mice/group for ND587 and ND610 and n= 6–7 mice/group for ND584, ND569, and ND627. For the spleen, n= 9, 12, and 12 mice for control M5CAR T cell, S CD3ζ&β2M M5CAR T cell, and KO CD3ζ&β2M M5CAR T cell groups, respectively. **(e)**, Dot plots of human CD45+ cells (first and second row) and M5CAR+ T cells (third row) detected in representative blood samples on day 26 after injection in S CD3ζ&β2M M5CAR T cell (S CD3ζ&β2M M5) or KO CD3ζ&β2M M5CAR T cell (KO CD3ζ&β2M M5) groups, from the same T cell donor. **(f, g)**, Dot plots of human CD45+ cells (first and second row) and M5CAR+ T cells (third row) analyzed in representative spleen samples on day 66 after injection in S CD3ζ&β2M M5CAR T cell [S CD3ζ&β2M M5; **(f)**], or KO CD3ζ&β2M M5CAR T cell [KO CD3ζ&β2M M5; **(g)**] groups, each compared with their respective donor-derived control M5CAR T cells (M5). For **(e, f, g)**, first and second rows are dot plots showing the expression of TCR (via CD3ϵ) and MHC-I (via β2M), respectively, along with M5CAR (M5), gated on the human CD45+ cell population. The third row shows dot plots of CD3ϵ and β2M expression gated on the M5CAR+ T cell population. Each symbol represents a different T cell donor (◼ ND587; ▲; ND610; ▼ND584; ◓ ND569; ⬣ ND627). P values (*p ≤ 0.05; **p ≤ 0.01) were determined by Wilcoxon Mann-Whitney test. In [**(c, d)** spleen], non-parametric tests were used for statistical analysis, but the small sample size prevented statistical significance-driven conclusions. Error bars represent mean (SD).

Differences in antitumor effects between treatment groups may be attributed to variations in the *in vivo* persistence of CAR T cells and/or M5CAR expression levels. On day 16, M5CAR+ T cells were detectable in 94.1% (32/34) of mice in the control M5CAR T cell group, 61.1% (22/36) in the S CD3ζ&β2M M5CAR T cell group, and only 22.9% (8/35) in the KO CD3ζ&β2M M5CAR T cell group. By day 26 of the second experiment, all mice (18/18) in the control M5CAR T cell group, 84.2% (16/19) in the S CD3ζ&β2M M5CAR T cell group, and just 26.3% (5/19) in the KO CD3ζ&β2M M5CAR T cell group still had detectable M5CAR+ T cells in circulation. Across both time points, CD4+ and CD8+ M5CAR+ T cell levels were highest in the control group, with the S CD3ζ&β2M M5CAR T cell group generally maintaining higher levels than the KO CD3ζ&β2M M5CAR T cell group (**Figures 8a, b**).

On day 66 of the second experiment, M5CAR+ T cells remained detectable in the spleens of all mice in the control M5CAR T cell group. Consistent with blood samples, splenic levels of adoptively transferred T cells were lower in both the S CD3ζ&β2M and KO CD3ζ&β2M M5CAR T cell groups (**Figure 8c**). In the S CD3ζ&β2M M5CAR T cell group, 75.0% (9/12) of mice had appreciable levels of M5CAR+ T cells, whereas only 33.3% (4/12) in the KO CD3ζ&β2M M5CAR T cell group had detectable levels. Differences in M5CAR expression levels observed *in vitro* were confirmed *in vivo*. Among mice with detectable M5CAR+ T cells, the MFI of S CD3ζ&β2M M5CAR+ T cells was lower than that of KO CD3ζ&β2M and control M5CAR+ T cells in both blood (days 16 and 26) and spleen (day 66) (**Figure 8d**).

Gene silencing in S CD3ζ&β2M M5CAR+ T cell population remained stable *in vivo*. On day 26, complete silencing of both target genes was still observed in the blood (**Figure 8e**) while the percentage of dual KO cells in the KO CD3ζ&β2M M5CAR+ T cell population had significantly declined (**Figure 8e**), averaging only 21.8% (10.1). Silencing persisted in the spleen on day 66, with 88.9% (8/9) of mice in the S CD3ζ&β2M M5CAR T cell group retaining full silencing of both CD3ζ and β2M (**Figure 8f**) while the KO CD3ζ&β2M M5CAR+ T cell group had largely lost the KO (**Figure 8g**). Among the four mice in the KO CD3ζ&β2M M5CAR T cell group with detectable M5CAR+ T cells, the majority of M5CAR+ T cells were non-KO, 64.9% (33.1), while only 18.8% (20.1) were dual KO, and 14.5% (14.7) retained a single CD3ζ KO.

Our findings indicate that S CAR T cells exhibited superior *in vivo* persistence compared to KO CAR T cells, despite having a lower percentage of M5CAR+ T cells and lower CAR MFI. The S CD3ζ&β2M M5CAR+ T cells maintained stable gene silencing in both blood and spleen. In contrast, the proportion of KO CD3ζ&β2M M5CAR^+^ T cells markedly decreased in the blood, and this reduction was subsequently confirmed in the spleen. These results demonstrate that S M5CAR+ T cells persist as a relatively homogeneous population, whereas KO M5CAR+ T cells become more heterogeneous over time. Control M5CAR T cells had the highest persistence and antitumor efficacy of all groups, suggesting that TCR absence in CAR T cells may impact potency and warrants further investigation.

### S CD3ζ&β2M M5CAR T cells exhibit greater resistance to allogeneic NK cell killing compared to KO CD3ζ&β2M M5CAR T cells, while similarly preventing alloreactivity from PBMCs

**Figure 9:**
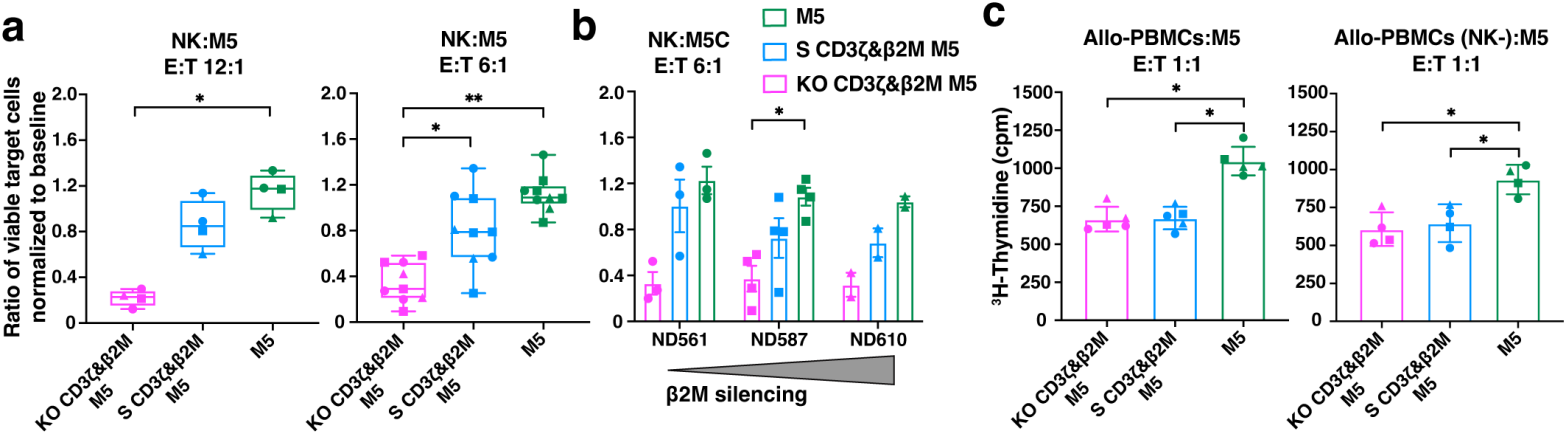
Higher protection of S CD3ζ&β2M M5CAR T cells against allogeneic NK cells than KO CD3ζ&β2M M5CAR T cells, with similar alloreactivity prevention from allogeneic PBMCs. **(a, b)** Live CalceinAM^high^ expressing TCRαβ-depleted silenced (S) and knockout (KO) M5CAR T target cells were assessed after a 4-hour co-culture with allo-NK cells at NK:T ratios of 12:1 **(a)** and 6:1 **(b)**. Data are presented as the ratio of the percentage of viable target cells in the co-culture condition to the percentage of viable target cells at baseline. In **(b)**, protection from allo-NK was correlated to the levels of MHC-I (via β2M) silencing across different TCRαβ-depleted M5CAR T cell donors. **(c)**, Proliferation of allo-PBMC responders, with or without NK cells, measured by ^3^H-thymidine incorporation after six days of co-culture with irradiated TCRαβ-depleted M5CAR T cells as stimulators at a 1:1 E:T ratio. Each point in the graphs represents an independent experiment. Three M5CAR T cell donors (◼ ND587; ▲; ND610; ● ND561) and four NK cell donors (for the 12:1 ratio) and nine NK cell donors (for the 6:1 ratio) were used in panel **(a)**. Three M5CAR T cell donors (◼ ND587; ▲; ND610; ● ND561) and three allo-PBMC donors (for conditions with NK cells) or two allo-PBMC donors (for conditions without NK cells) were used in panel **(c)**. P values (*p ≤ 0.05; **p ≤ 0.01) were calculated by Wilcoxon Mann-Whitney test. Error bars represent median (IQR) **(a)** and mean (SD) **(b, c)**.

To evaluate the immunogenicity of S CD3ζ&β2M M5CAR T cells and their resistance to host immune responses, we conducted co-culture assays with alloreactive NK cells and peripheral blood mononuclear cells (PBMCs). TCRαβ-depleted M5CAR T cells were co-cultured with freshly isolated peripheral blood NK cells from non-matched healthy donors. S CD3ζ&β2M M5CAR T cells, which retain partial MHC-I expression, were significantly less immunogenic to allogeneic NK cells compared to KO CD3ζ&β2M M5CAR T cells, although not to the extent observed in M5CAR T cells fully expressing MHC-I (**Figure 9a**). The level of protection from allo-NK cells appeared to be inversely correlated with the degree of MHC-I silencing across different M5CAR T cell donors (**Figure 9b**).

Next, we assessed whether MHC-I silencing in S CD3ζ&β2M M5CAR T cells was sufficient to prevent recognition and activation of allogeneic PBMCs (allo-PBMCs). As expected, the strongest allo-PBMC response was observed when stimulated by control M5CAR T cells (**Figure 9c**). Both S CD3ζ&β2M and KO CD3ζ&β2M M5CAR T cells exhibited a similarly reduced stimulatory capacity of allo-PBMCs, regardless of the presence of NK cells (**Figure 9C**). These data suggest that the tuned low surface MHC-I expression provides better protection against NK cell-mediated killing for S CD3ζ&β2M M5CAR T cells compared to KO CD3ζ&β2M M5CAR T cells. Additionally, functional MHC-I silencing in S CD3ζ&β2M M5CAR T cells was sufficient to prevent alloreactive responses from allo-PBMCs, comparable to that observed in KO CD3ζ&β2M M5CAR T cells.

## Discussion

Safe and effective multiplex gene engineering is needed for enhancement and diversification of CAR T cell therapeutics. This study assessed miRNA-mediated gene silencing as an alternative to CRISPR/Cas9 gene editing for multiplex engineering of CAR T cells in a solid tumor model.

Several modalities, including RNA interference (RNAi), antisense oligonucleotides, and epigenome editing are currently being explored to silence genes without altering DNA sequences (21, 22). Small interfering RNA (siRNA) has been particularly successful, securing six FDA approvals (23). While siRNA is highly specific and potent, its temporary effects are not suitable for gene therapies that require permanent gene silencing. To this end, genome integration techniques involving miRNA or short hairpin RNA (shRNA) constructs have been developed to enable continued target gene silencing (15, 24, 25). Unlike CRISPR-based gene editing, BE or PE, which result in permanent changes to DNA, miRNA and shRNA-mediated silencing modulates target gene expression at the post-transcriptional level. This approach has been well established and clinically translated by several groups in the past (26–28). A notable advantage of miRNA over shRNA is that multiple genes can be targeted from a single gene construct, while shRNA constructs require that each shRNA is expressed from its own pol III promoter. Also, while canonical miRNA expression follows natural processing by Drosha and Dicer enzymes, shRNA artificially bypasses Drosha, potentially oversaturating the endogenous RNAi machinery and thus risking acute cellular toxicity (29–31). Consequently, several groups have adopted an approach of developing “miRNA-embedded” shRNA constructs in an attempt to mitigate the toxicity risks (15, 25, 28).

The optimized miRNA design (mirGE) employed in this study offers high fidelity, multiplexing capabilities and tunable expression, making it a versatile alternative to enhance CAR T cell functionality and applicability (14, 32, 33).

Here, as a proof of concept to assess the robustness of miRNA-mediated gene silencing versus CRISPR/Cas9-mediated gene deletion in manufacturing engineered CAR T cells, non-alloreactive, hypoimmunogenic CAR T cells were generated using both approaches. Gene inhibition and subsequent T cell performance were evaluated in a PDAC solid tumor model.

Building on the safety of vector-engineered CAR T cells in clinical applications for HIV and cancer (34–36), dual-targeting miRNAs against CD3ζ to abrogate TCR expression, and a single miRNA against β2M for tuned MHC-I silencing were selected and incorporated into a bimodal lentiviral construct also coding the M5CAR gene. Importantly, this enabled simultaneous expression of miRNAs and M5CAR, achieving multiplex functionality in CAR T cells through a single transduction step, eliminating the need for the second manufacturing step of EP or NF as required for CRISPR/nuclease-mediated editing, BE or PE.

The manufacturing process using miRNAs produced higher yields and phenotypic profiles comparable to control CAR T cells, whereas the CRISPR/Cas9 KO process resulted in lower yields and a less desirable differentiated phenotype, likely due to the NF step and decreased expansion. These potentially concerning limitations in KO-based manufacturing could be mitigated by alternative methods for delivering ribonucleoproteins without EP or NF, which are currently being evaluated to reduce T cell toxicity (37–40). Another benefit of manufacturing modified CAR T cells in a single step using miRNA constructs is the uniform purity of the final product. After TCRαβ depletion, nearly all cells were miRNA-S and CAR+. In contrast, TCRαβ-depleted CRISPR/Cas9-edited M5CAR T cells resulted in a heterogeneous population, consisting of CAR+ and non-transduced T cells, both carrying the KO, with a subset still expressing β2M. The proportions of CAR+ cells and β2M+ cells varied depending on the transduction and KO efficiencies, respectively. Therefore, the miRNA manufacturing process produces a more defined and purified therapeutic product.

*In vitro* experiments showed that the antitumor function of S CD3ζ&β2M M5CAR T cells was largely comparable to that of control M5CAR T cells. Both S CD3ζ&β2M M5CAR T cells and KO CD3ζ&β2M M5CAR T cells exhibited similarly impaired expansion during co-culture with tumor cells compared to control M5CAR T cells, though the extent varied among T cell donors. This effect was more pronounced in CAR+ T cells than in non-transduced T cells, likely due to defects in expansion and/or an increased tendency for CAR downregulation following antigen stimulation, as previously observed (17).

*In vivo*, S CD3ζ&β2M M5CAR T cells demonstrated superior persistence compared to KO CD3ζ&β2M M5CAR T cells in the majority of T cell donors tested. This enhanced persistence was associated with a trend toward better control of primary tumors and, notably, improved prevention of metastatic spread. Importantly, silencing levels in S CD3ζ&β2M M5CAR T cells remained stable and uniform for both target genes, confirming previous findings (14). In contrast, the KO CD3ζ&β2M M5CAR T cell group exhibited heterogeneous populations, indicating that non-purified manufactured material persisted, potentially due to detrimental effects of multiplex gene editing, which promoted the survival of less-edited cells. For clinical applications of engineered cell therapy, regulatory compliance and quality control considerations favor more homogeneous products, making miRNA-based gene silencing a promising strategy. Control M5CAR T cells demonstrating superior antitumor effects and persistence *in vivo* compared to modified groups. While loss of TCR signaling may contribute to reduced potency in this system, by limiting tonic or bystander activation, it is unlikely to be the sole factor. Although CAR and TCR signal independently, their co-localization in membrane microdomains may enhance immune synapse formation and signal amplification via shared mediators such as Lck. The critical role of the endogenous TCR in sustaining long-term responses was confirmed with CAR19 T cells, where CRISPR/Cas9-mediated TCR KO impaired persistence *in vivo* (41). Notably, this effect might be amplified in mouse models, as human TCRs can cross-react with murine MHC molecules, potentially enhancing T cell expansion and persistence (42). Indeed, we found that most mice receiving control CAR T cells developed xenogeneic GvHD, likely favored by such a cross-reaction. In addition to TCR loss, S M5CAR T cells exhibited a lower percentage of CAR-expressing cells and reduced CAR MFI compared to both control and KO groups, likely impairing antigen-driven activation and tumor control *in vivo*. This reduced expression is attributed to the increased size and complexity of the miCAR construct, which may compromise lentiviral packaging, transduction efficiency, and transcriptional output. While this may slightly reduce potency, the functional benefit conferred by the silencing moieties supports the clinical relevance of miRNA-based silencing in CAR T cell therapies.

Deficiencies in expansion and/or downregulation of the CAR may have also occurred in S and KO CAR T cells *in vivo* (17), with the possibility that downregulation could be beneficial if surface expression is restored for improved persistence. Further exploration is needed to identify signs of premature dysfunction, which may affect both *in vitro* persistence and *in vivo* performance.

The complete ablation of certain targets via gene editing can in some circumstances be detrimental, whereas tunable miRNA-based silencing may offer a better balance. For example, significantly though not completely downregulated MHC-I is preferable to its complete loss, which makes engineered cells vulnerable to NK cell-mediated lysis, requiring compensatory strategies like overexpression of the HLA class I histocompatibility antigen, alpha chain E (HLA-E) (43). The hypothesis that tunable silencing of MHC-I would render non-alloreactive S CD3ζ&β2M M5CAR T cells hypoimmunogenic was confirmed in co-culture assays with allogeneic NK and PBMCs. A stable low MHC-I density on the cell surface provided better protection for S CD3ζ&β2M M5CAR T cells from ‘missing self’-induced NK cell killing compared to KO CD3ζ&β2M M5CAR T cells. Similarly, Rossi et al., selected a miRNA against β2M to allow for residual MHC-I expression, limiting NK cell activation to levels comparable to those measured in presence of CAR T cells with no miRNA (15). Additionally, the MHC-I expression level on S CD3ζ&β2M M5CAR T cells was sufficiently low to prevent alloreactivity by allo-PBMCs, comparable to KO CD3ζ&β2M M5CAR T cells.

For the clinical translation of ‘off-the-shelf’ CAR T cells, several clinical studies using TALEN, CRISPR/Cas9 or BE for the removal of TCRαβ combined with depletion of residual TCRαβ-expressing cells, have proven to be a successful mitigation of GVHD, resulting in absence or limited severity of GVHD which was readily managed with immunosuppression (5–7, 44). Moreover, CAR T cells incorporating a shRNA-based cassette for the silencing of CD3ζ were proven safe in clinical trials (44, 45).

Beyond the benefit of tuned MHC-I silencing, the true potential of gene silencing lies in the ability to regulate expression levels of genes that are essential for antitumor T cell functions and homeostasis but require careful modulation to avoid adverse effects. Genes like TOX (46), which are upregulated following antigen stimulation, can drive T cell dysfunction when excessively expressed, while their complete deletion may lead to harmful activation-induced cell death, particularly in chronic antigen environments. Optimizing miRNAs for tuned gene silencing can prevent both overexpression-induced dysfunction and the risks associated with gene deletion, ultimately enhancing the durability and effectiveness of CAR T cell therapy. Future advancements could include expressing miRNAs under signal-activated or repressible promoters, enabling reversible or controlled silencing, thereby unlocking the full potential of miRNA-treated CAR T cells (22).

Nevertheless, several limitations should be acknowledged. First, we did not evaluate or compare potential off-target effects of miRNA- and CRISPR/Cas9-mediated editing, leaving the safety profiles of both approaches only partially explored. Second, donor-to-donor variability contributed to differences in T cell performance, highlighting the need for broader validation across multiple donors. Third, *in vivo* studies were conducted in non-humanized mouse models, which do not fully recapitulate interactions with a human immune system. Further studies will be important to validate and extend these findings in more physiologically relevant settings.

The development of efficiently multiplex-engineered potent CAR T cells may offer potential solutions to overcome resistance in tumors and address limitations associated with autologous therapies. Our findings demonstrate the feasibility of multiplex gene silencing and simultaneous CAR expression in T cells through a single lentiviral vector transduction event. The simplicity of this approach enables the convenience of silencing several genes without relying on multiple independent edits and risking chromosomal rearrangements and genomic instability. Broadening the spectrum of target genes for silencing, including those involved in CAR T cell suppression, fitness, and potency, could further enhance functionality and efficacy of miRNA-S CAR T cells against challenging tumors.

## Data Availability

The original contributions presented in the study are included in the article/**Supplementary Material**. Further inquiries can be directed to the corresponding authors.
