# Supplementary material for: Multiplex engineering using microRNA-mediated gene silencing in CAR T cells
Source: Front Immunol. 2025 Aug 21;16:1647433. doi: 10.3389/fimmu.2025.1647433 (PMC12408270; doi:10.3389/fimmu.2025.1647433)
Supplement: Supplementary file 1 [file DataSheet1.pdf]

Supplementary Material

Supplementary Figures and Tables

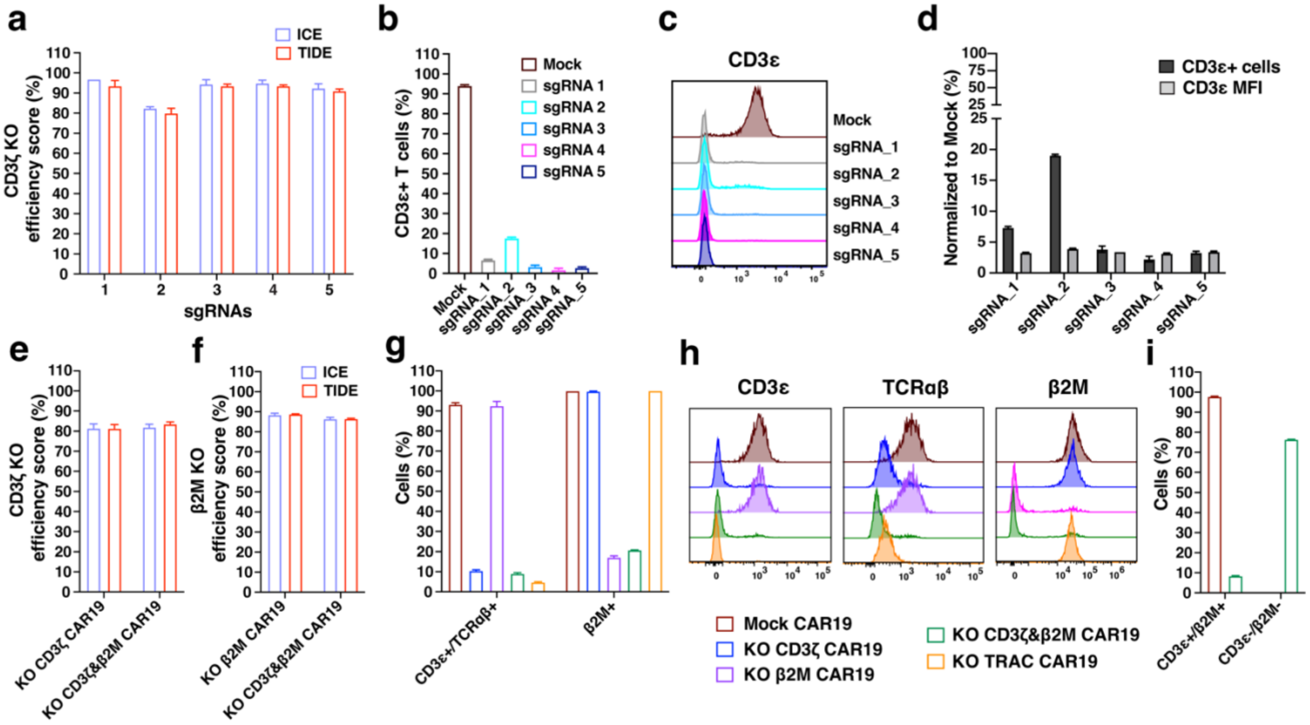

Supplementary Figure 1. Screening and selection of sgRNAs for CD3ζ deletion and evaluation of dual CD3ζ and β2M deletion in CAR19 T cells. (**A**), Efficiency score percentage for the knockout (KO) of the CD3ζ target gene using the screened sgRNAs, from 1 to 5. (**B**), Percentage of CD3ε-expressing T cells after KO with the indicated sgRNAs and in control Mock-edited T cells. (**C**), Representative histograms reporting CD3ε expression levels in T cells after KO with the indicated sgRNAs. (**D**), Percentage of CD3ε-expressing T cells and CD3ε median fluorescence intensity (MFI) in T cells after KO with the indicated sgRNAs, all normalized to control Mock T cells. sgRNA_4 was selected due to its high KO efficiency score resulting in only 2.1 (0.4) % CD3ε+ T cells with the lowest MFI. (**E** and **F**), Efficiency score percentage for the KO of the CD3ζ (E) or β2M (F) target genes alone or simultaneously in CAR19 T cells. (**G**), Percentage of remaining CD3ε+/TCRαβ+ or β2M+ cells in the different CAR19 T cell groups. (**H**), Representative histograms showing CD3ε, TCRαβ, and β2M expression levels after KO in the different CAR19 T cell groups. (**I**), Percentage of remaining dual CD3ε+/β2M+ and dual CD3ε-/β2M- cells in KO CD3ζ&β2M CAR19 T cells (KO CD3ζ&β2M CAR19 T cells) compared to Mock CAR19 T cells. Simultaneous CD3ζ and β2M deletions in KO CD3ζ&β2M CAR19 T cells were comparable to single-target deletions. The proportions of remaining CD3ε+/TCRαβ+ or β2M+ cells, as well as the MFI of surface CD3ε/TCRαβ and β2M in KO CD3ζ&β2M CAR19 T cells, were as low as those observed in single CD3ζ or β2M KO conditions, respectively. The CD3ε/TCRαβ levels in KO CD3ζ&β2M CAR19 T cells were also similar to those in

CAR19 T cells where TCR deletion was achieved by targeting the conventional TRAC gene. In KO CD3ζ&β2M CAR19 T cells, the remaining dual CD3ε+/β2M+ cells accounted for 0.4 (0.2) % of total cells, while dual CD3ε-/β2M- cells constituted 76.5 (0.1) % of total cells. According to Tracking of Indels by Decomposition (TIDE) predictions, over 70% of indels in both CD3ζ and β2M were deletions, predominantly consisting of single-nucleotide deletions. N= 1 T cell donor/condition with at least two technical replicates for each graph. The small sample size prevented statistical significance-driven conclusions. Error bars represent mean (SD).

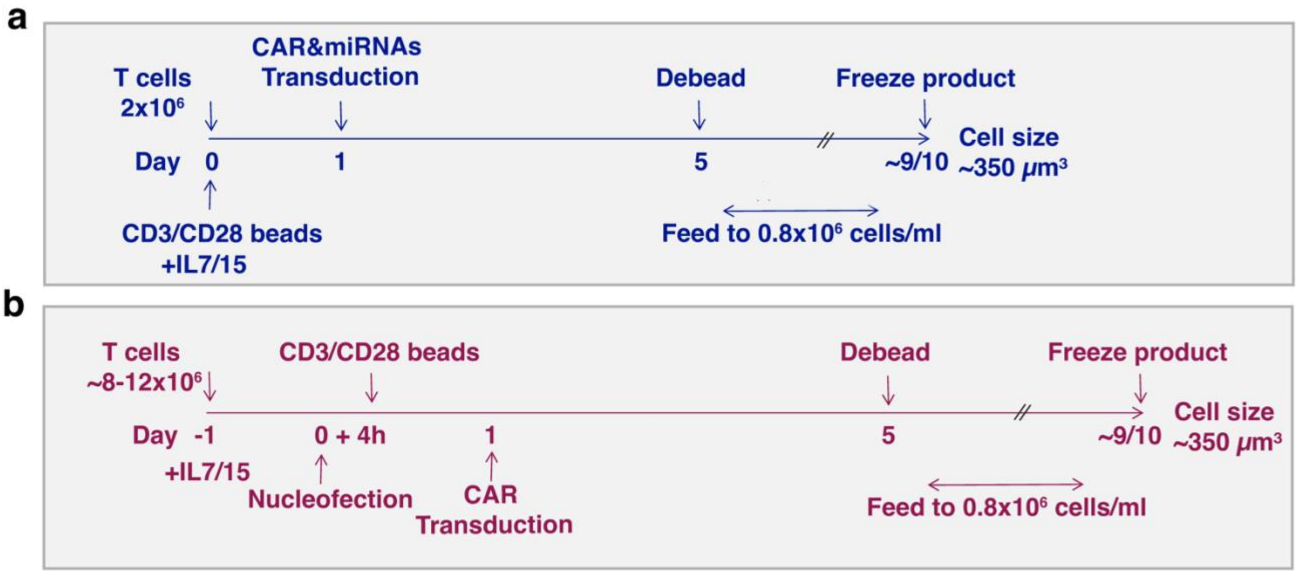

Supplementary Figure 2. Production of M5CAR T cells with silencing or knockout of CD3ζ and β2M target genes. (**A**), T cells undergoing miRNA-based silencing were activated using CTS™ Dynabeads™ CD3/CD28 (day 0). Lentiviral vectors coding for the M5CAR and CD3ζ - and/or β2M-targeting miRNAs were used for transduction approximately 24 hours post bead stimulation (day 1). For each silencing condition, 2 x 10^6 cells were transduced, as in the control M5CAR T cell group. (**B**), T cells undergoing the CRISPR/Cas9-genome engineering were incubated overnight with IL-7 and IL-15 (day -1). Next day, T cells were nucleofected with Cas9 and sgRNA ribonucleoprotein complexes (day 0). For each knockout condition, approximately 8-12 x 10^6 cells were nucleofected. Four hours later, nucleofected cells were then activated using CTS™ Dynabeads™ CD3/CD28. After 24 hours, all counted cells were transduced with the M5CAR lentiviral vector (day 1). Over both protocols (A, B), beads were removed on day 5 of stimulation. Cells were then monitored daily until growth kinetics and cell size demonstrated that they had rested from stimulation. On average, T cells were grown for nine or ten days in the presence of IL-7 and IL-15 and maintained at 8 x 10^5 cells/mL prior to cryopreservation.

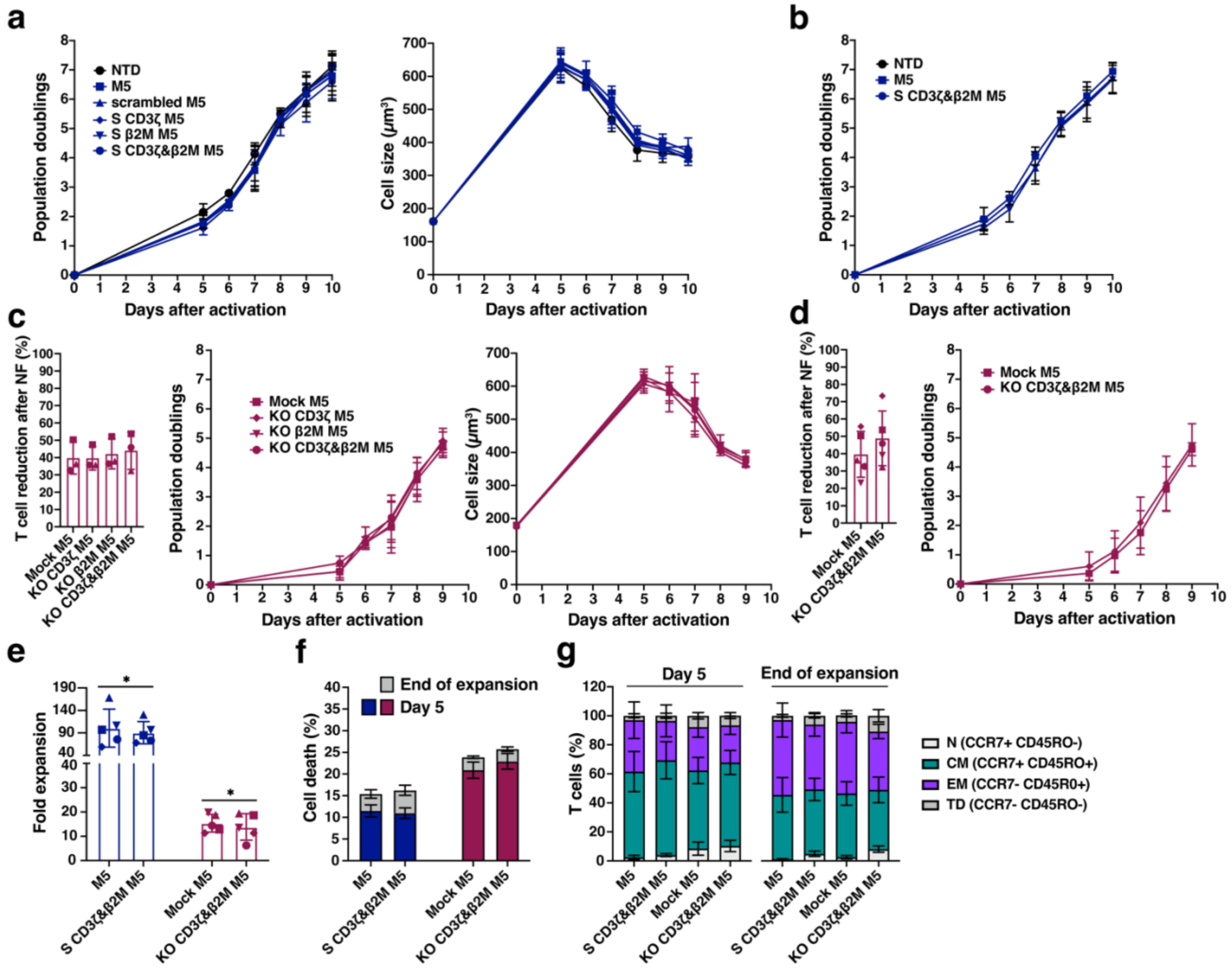

Supplementary Figure 3. Higher yield of expansion, viability, and preservation of memory phenotypes in silenced M5CAR T cells versus CRISPR/Cas9-edited ones. (**A**) to (**D**), Population doublings (PDs) and cell size evaluated over expansion. PDs were calculated as the $\log_2$ of the ratio of the final number of cells to the initial number of cells. For KO M5CAR T cell groups, the cell number after nucleofection (NF) was used as the initial value. The percentage reduction in T cell numbers after NF is also reported for the KO M5CAR T cell groups (C and D), defined as the difference between pre- and post-NF cell counts, divided by the pre-NF count and multiplied by 100. Not transduced T cells, NTD; M5CAR T cells, M5; M5CAR T cells with scrambled miRNA (scrambled M5); M5CAR T cells with miRNAs for single-silenced (S) CD3ζ (S CD3ζ M5) or β2M (S β2M M5) transcripts; M5CAR T cells with miRNAs for combined silencing of CD3ζ and β2M (S CD3ζ&β2M M5); Mock-edited M5CAR T cells (Mock M5); M5CAR T cells with sgRNAs for single-knockout (KO) of CD3ζ (KO CD3ζ M5) or β2M (KO β2M M5) genes; M5CAR T cells with combined sgRNAs for dual KO of CD3ζ and β2M genes (KO CD3ζ&β2M M5CAR). PDs of both single and dual CD3ζ and β2M S M5CAR T cell groups were comparable to those of the control M5CAR T cells, with mean (SD) values of 6.54 (0.44) (A) and 6.61 (0.44) (B) for the S CD3ζ&β2M M5CAR T cell group. As for KO M5CAR T cells, the cells almost halved after NF (C and D). Considering the cell count after NF as the initial number, PDs for KO cells were significantly lower than those of S cells, with mean (SD) PD values of 4.84 (0.49) (C) and 4.67 (0.80) (D) for the KO CD3ζ&β2M M5CAR T cell group. (**E**), Fold expansion of M5CAR T cells expressed as the ratio of the final number of cells to the initial number of cells.

Starting with 2 x 10^6 T cells for both S and control M5CAR T cell conditions, the S CD3ζ&β2M M5CAR T cell group achieved a mean (SD) amount of 181 x 10^6 (50 x 10^6) T cells, which was comparable to that of the M5CAR T cell group. In KO groups, which started with a higher mean of 10.5 x 10^6 (1.6 x 10^6) T cells, numbers halved after NF, 5.87 x 10^6 (1.12 x 10^6) cells, reaching 149 x 10^6 (68 x 10^6) T cells in the KO CD3ζ&β2M M5CAR T cell group at the end of expansion. This was similar to the Mock M5CAR T cell group. (**F**), Cell death of M5CAR T cells on day 5 and at the end of expansion. (**G**), T cell memory subsets in M5CAR+ T cells by CD45RO and CCR7 markers on day 5 and at the end of expansion. Naïve-like, N; Central memory, CM; effector memory, EM; terminally differentiated, TD. N= 3 (A and C) and 5 (B, D, E, F, and G) T cell donors/group. In (E), each symbol represents a different T cell donor (■ ND587; ▲ ND610; ● ND561; ◆ ND365; ▼ND584). P value (*$p \leq 0.05$) was determined by Wilcoxon Mann-Whitney test. In (F, G), non-parametric tests were used for statistical analysis, but the small sample size prevented statistical significance-driven conclusions. Error bars represent mean (SD) (A, B, C, D, E) and mean (SEM) (F, G).

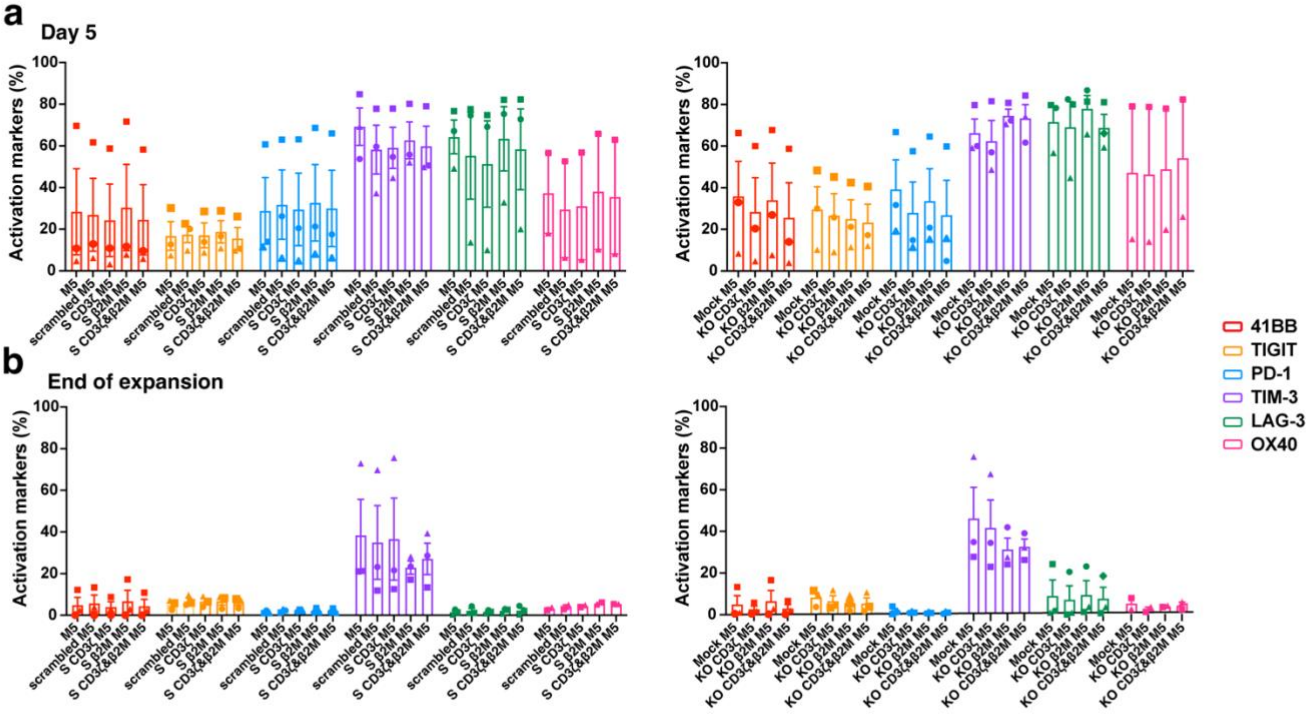

Supplementary Figure 4. Expression of T cell activation markers on day 5 and at the end of M5CAR T cell expansion. (**A**) and (**B**), Silenced (S) and knockout (KO) M5CAR+ T cells were compared in terms of expression levels of 41BB, TIGIT, PD-1, TIM-3, LAG-3, and OX40 activation markers on day 5 (A) and at the end of cell culture expansion (B). N= 2-3 T cell donors/group. Each symbol represents a different T cell donor (■ ND587; ▲ ND610; ● ND561). Non-parametric tests were used for statistical analysis, but the small sample size prevented statistical significance-driven conclusions. Error bars represent mean (SD).

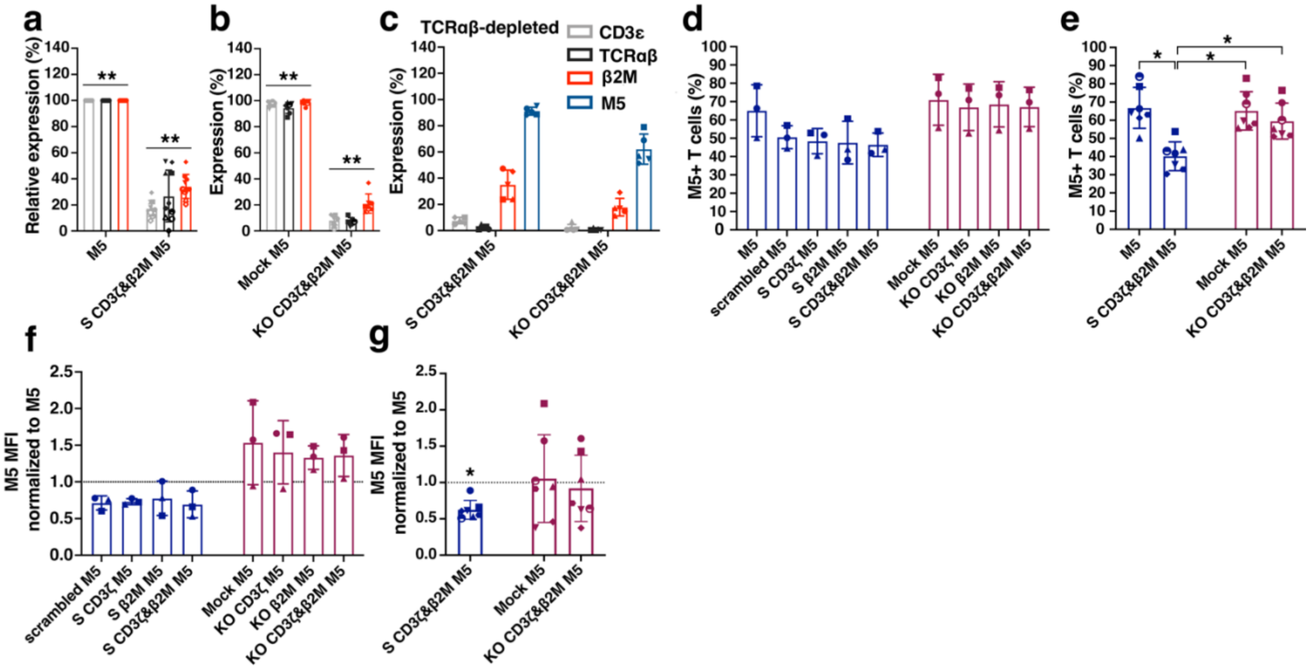

Supplementary Figure 5. Comparing multiplex silencing and deletion of TCR and MHC-I and the efficiency of M5CAR expression in modified M5CAR T cells. (**A**), Relative expression of TCR (via CD3ε and TCRαβ) and MHC-I (via β2M) in silenced (S) CD3ζ&β2M M5CAR T cells at the end of expansion, normalized to control M5CAR T cells. (**B**), Expression of TCR and MHC-I on knockout (KO) CD3ζ&β2M M5CAR T cells at the end of expansion. (**C**), Expression of TCR and MHC and M5CAR after TCRαβ depletion in S CD3ζ&β2M M5CAR T cells and KO CD3ζ&β2M M5CAR T cells. The mean (SD) yield of TCRαβ depletion was slightly, but not significantly, lower for S CD3ζ&β2M M5CAR T cells, 4.1% (13.5), compared to KO CD3ζ&β2M M5CAR T cells, 75.9% (25.3). (**D**) and (**E**), Percentage of M5CAR+ T cells over total T cells in S, KO, control, and Mock M5CAR T cells. (**F**) and (**G**), Normalized median fluorescence intensity (MFI) of M5CAR in the M5CAR+ T cell population compared to control M5CAR T cells. N= 10 (A), 7 (B, E, G), 5 (C), 3 (D, F) T cell donors/group. Each symbol represents a different T cell donor (■ ND587; ▲ ND610; ● ND561; ◆ ND365; ▼ND584; ◓ ND569; ⬣ ND627; ◇ ND541; ○ ND224; △ ND582). P values (*$p \leq 0.05$) were calculated by Wilcoxon Mann-Whitney test. In (D, F), non-parametric tests were used for statistical analysis, but the small sample size prevented statistical significance-driven conclusions. Error bars represent mean (SD).

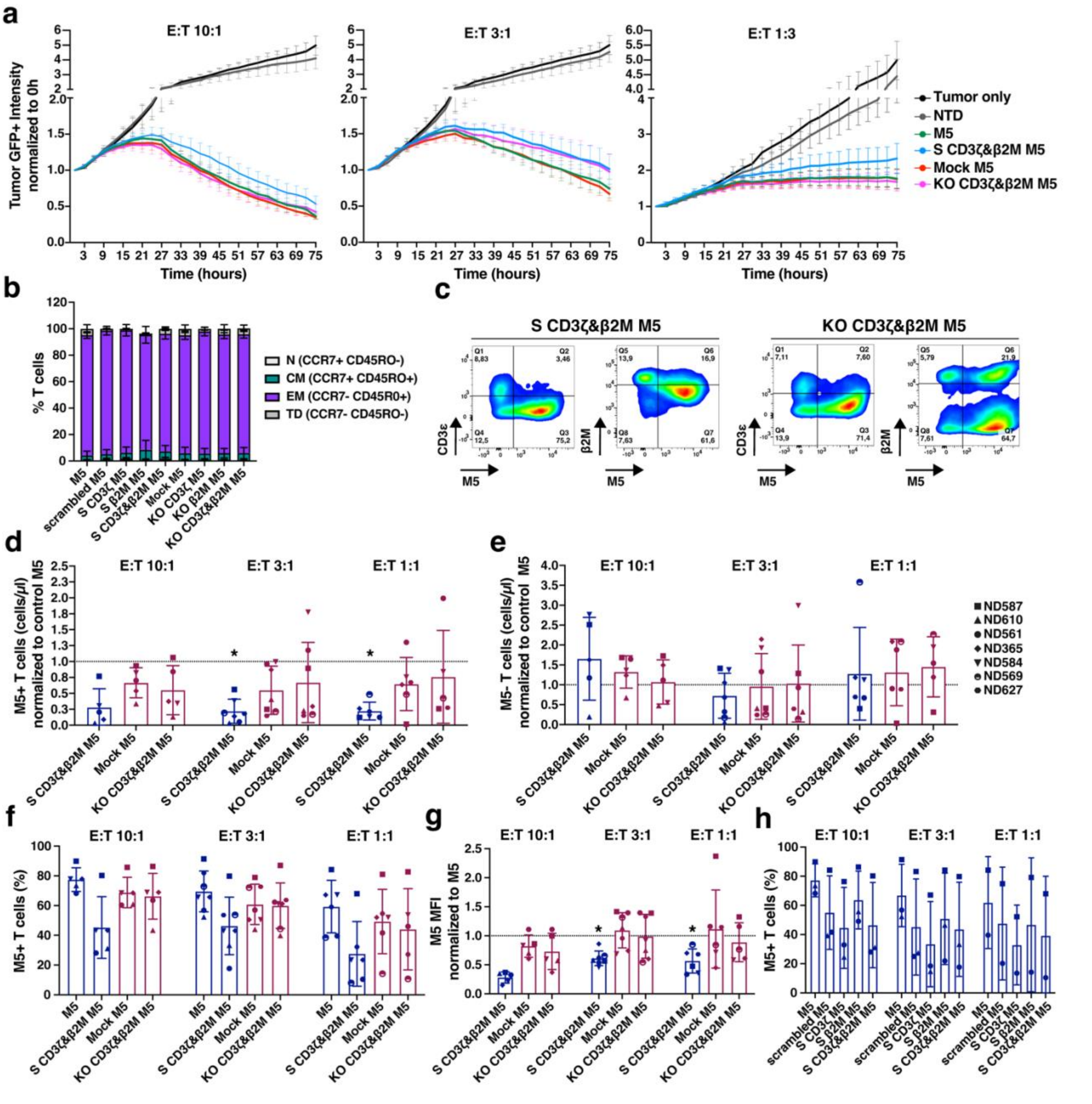

Supplementary Figure 6. Evaluating cytotoxicity, expansion and M5CAR expression in TCR and MHC-I silenced versus knockout M5CAR T cells after long-term co-culture. (**A**), Viability of AsPC-1 tumor cells after co-culture with silenced (S) and knockout (KO) M5CART cells at effector:target (E:T) ratios of 10:1, 3:1, and 1:3, evaluated in real-time over more than 3 days of co-culture. (**B**), T cell memory subsets in M5CAR+ T cells of ND587, ND610, and ND561 T cell donors by CD45RO and CCR7 markers after six days of co-culture with AsPC-1 at the 10:1 E:T ratio. Naïve-like, N; Central memory, CM; effector memory, EM; terminally differentiated, TD. (**C**), Dot plots of M5CAR T cells from representative samples collected after six days of co-culture between ND587 S CD3ζ&β2M M5CAR T cells (S CD3ζ&β2M M5) or KO CD3ζ&β2M M5CAR T cells (KO CD3ζ&β2M M5) and AsPC-1 at the 3:1 E:T ratio. (**D**) and (**E**), Ratio of the concentration (cells/µl) of

M5CAR+ (A) and M5CAR- (B) CAR T cells quantified after six days of co-culture with AsPC-1 at the indicated E:T ratios and normalized by the control M5CAR T cell group. (**F**) to (**H**), Percentage of M5CAR+ T cells over total live cells in co-culture (F and H) and normalized median fluorescence intensity (MFI; G) of the M5CAR in the M5CAR+ T cell population compared to the control M5CAR T cell group after six days of co-culture with AsPC-1 at the indicated E:T ratios. N= 7 (A), 3 (B and H), 5-7 (D to G) T cell donors/group. Each symbol represents a different T cell donor (■ ND587; ▲ ND610; ● ND561; ◆ ND365; ▼ND584; ◓ ND569; ⬣ ND627). P value (*$p \leq 0.05$) was calculated by Wilcoxon Mann-Whitney test. In (H), non-parametric tests were used for statistical analysis, but the small sample size prevented statistical significance-driven conclusions. Error bars represent mean (SD) (A, D, E, F, G, and H) and mean (SEM) (B).

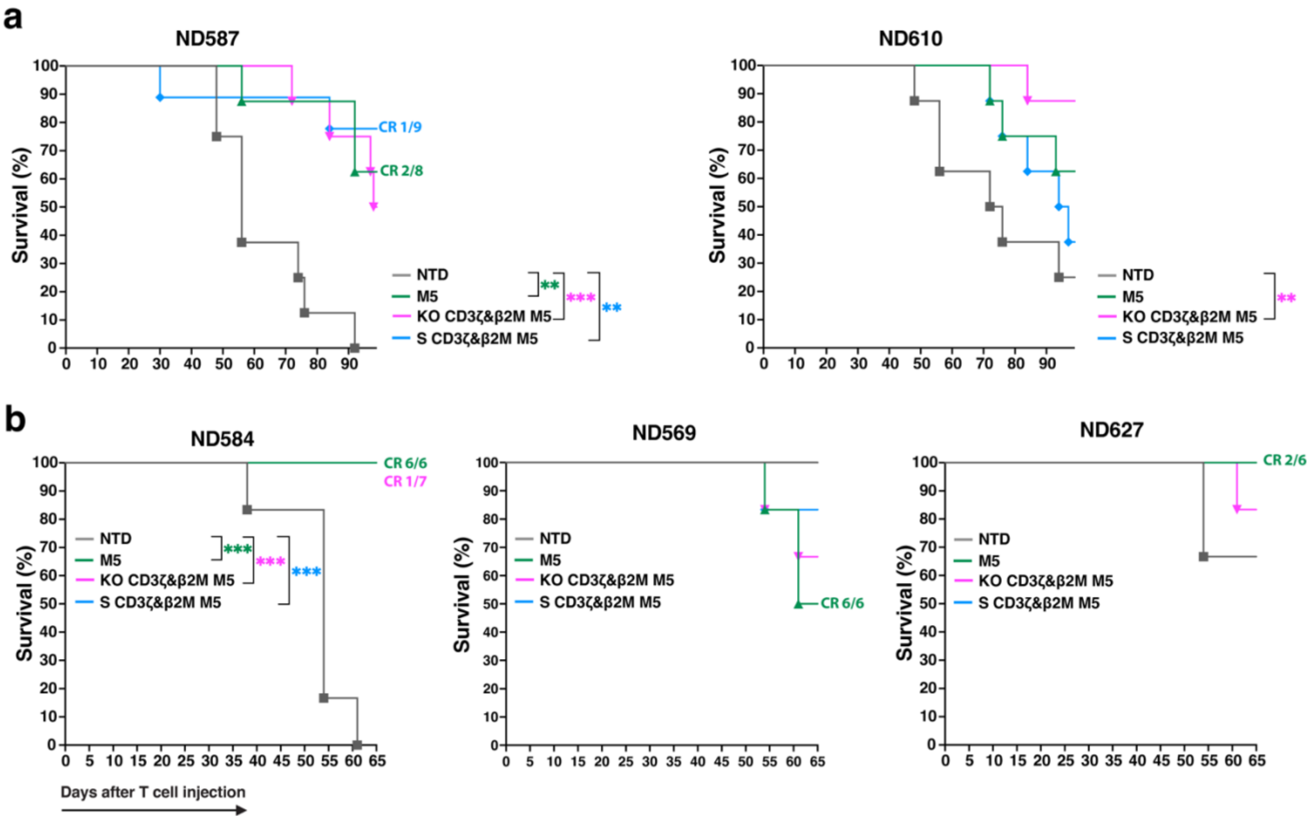

Supplementary Figure 7. Survival of mice treated with S CD3ζ&β2M M5CAR T cells versus KO CD3ζ&β2M M5CAR T cells. (**A**) and (**B**), Kaplan-Meier survival curves from two independent *in vivo* experiments using T cells from five different donors. The first experiment included donors ND587 and ND610 (n = 8–9 mice/group per donor); the second included donors ND584, ND569, and ND627 (n = 6–7 mice/group per donor). Mice were euthanized per protocol if tumor diameter exceeded 20 mm, if ulceration affected more than 90% of the tumor surface, or if body weight loss exceeded 20%. A fixed observation period was applied: 100 days for the first experiment and 66 days for the second, measured from the day of CAR T cell injection. CR, complete response, defined as the absence of palpable tumor in treated mice. P values (**$p \leq 0.01$; ***$p \leq 0.001$) were calculated by Kaplan Meier estimator and pairwise comparisons using the Log-Rank test.

Target Gene	Sequence ID	sgRNA sequence (5’ to 3’)
**CD3ζ**	CD3ζ sgRNA_1	GTGGAAGGCGCTTTTCACCG
	CD3ζ sgRNA_2	CGGCAACTGTGCCTGCAGGA
	CD3ζ sgRNA_3	AGCAGAGTTTGGGATCCAGC
	CD3ζ sgRNA_4	TGAAGAGGATTCCATCCAGC
	CD3ζ sgRNA_5	GAATGACACCATAGATGAAG
**β2M**	β2M sgRNA	GAGTAGCGCGAGCACAGCTA
**TRAC**	TRAC sgRNA	CGTCATGAGCAGATTAAACC

Supplementary Table 1. sgRNA sequences screened to select the best sgRNA for knockout of TCR and MHC-I complexes.

Target Gene	Sequence ID	miRNA sequence (5’ to 3’)
**CD3ζ**	**CD3ζ_T1**	GGTGATAGCAATGTCAGCAGTGCCTTTTGGAGCTAAATATAACCAAAGTGAAGCCACA<br>GATGTTTGGTTATACTTAGCTCCAACAAGTAAGGTTGACCATACTCTAC
	**CD3ζ_T2**	GGTGATAGCAATGTCAGCAGTGCCTTATCCTAGTACATTGACGGGTTGTGAAGCCACA<br>GATGAACCCGTCAACGTACTAGGATCAAGTAAGGTTGACCATACTCTAC
	CD3ζ_T3	GGTGATAGCAATGTCAGCAGTGCCTTTCCACTTCATCTTGTCCTTTCGTGAAGCCACAG<br>ATGGAAAGGACAAAATGAAGTGGACAAGTAAGGTTGACCATACTCTAC
	CD3ζ_T4	GGTGATAGCAATGTCAGCAGTGCCTTTTTATTCTGCAGCTACCTCCCGTGAAGCCACAG<br>ATGGGGAGGTAGCCGCAGAATAAACAAGTAAGGTTGACCATACTCTAC
	CD3ζ_T5	GGTGATAGCAATGTCAGCAGTGCCTAGGTTTGGAGCTAAATATAACCGTGAAGCCACA<br>GATGGGTTATATTTCGCTCCAAACCCAAGTAAGGTTGACCATACTCTAC
**β2M**	β2M_T1	GGTGATAGCAATGTCAGCAGTGCCTTAAATATCCATAGATTTGGTGTGTGAAGCCACAG<br>ATGACACCAAATCCATGGATATTTCAAGTAAGGTTGACCATACTCTAC
	β2M_T2	GGTGATAGCAATGTCAGCAGTGCCTTTGACTTTCCATTCTCTGCTGGGTGAAGCCACAG<br>ATGCCAGCAGAGACTGGAAAGTCACAAGTAAGGTTGACCATACTCTAC
	β2M_T3	GGTGATAGCAATGTCAGCAGTGCCTTTATGCACGCTTAACTATCTTAGTGAAGCCACAG<br>ATGTAAGATAGTTCAGCGTGCATACAAGTAAGGTTGACCATACTCTAC
	β2M_T4	GGTGATAGCAATGTCAGCAGTGCCTTTAAACAATAACAACTATTTGCGTGAAGCCACAG<br>ATGGCAAATAGTTATTATTGTTTACAAGTAAGGTTGACCATACTCTAC
	**β2M_T5**	GGTGATAGCAATGTCAGCAGTGCCTTAAACCTGAATCTTTGGAGTACGTGAAGCCACA<br>GATGGTACTCCAAAAATTCAGGTTTCAAGTAAGGTTGACCATACTCTAC

Supplementary Table 2. miRNA sequences screened to select the best miRNAs for silencing of TCR (via CD3ζ) and MHC-I (via β2M) complexes. Selected miRNA sequence IDs are in bold. Highlighted are the guide strand (blue text), the seed region (green highlight), the passenger strand (red text), and the loop region (underlined text).

	M5								
	ND584	ND569	ND627	ND561	ND587	ND610	ND365	Average	SD
CD4+ M5+ T cells	43.9	72.2	34.3	70.4	62.6	26.6	68.9	54.1	18.9
CD8+ M5+ T cells	47.1	24.3	62.0	28.1	31.5	60.6	20.5	39.2	17.3

	S CD3ζ&β2M M5								
	ND584	ND569	ND627	ND561	ND587	ND610	ND365	Average	SD
CD4+ M5+ T cells	46.9	73.2	37.2	73.7	67.2	28.0	58.3	54.9	18.0
CD8+ M5+ T cells	39.3	25.0	50.8	24.4	28.1	64.1	20.2	36.0	16.3

	KO CD3ζ&β2M M5								
	ND584	ND569	ND627	ND561	ND587	ND610	ND365	Average	SD
CD4+ M5+ T cells	49.6	67.1	29.9	63.7	44.9	36.2	61.2	50.4	14.3
CD8+ M5+ T cells	39.9	30.5	68.0	35.1	37.9	55.1	26.2	41.8	14.7

Supplementary Table 3. Variability among T cell donors in the percentage of CD4+ and CD8+ M5CAR+ T cells at the end of expansion across control (M5), dual CD3ζ and β2M silenced (S), and dual CD3ζ and β2M knockout (KO) M5CAR T cell groups.

Donor	Group	M5+ Expansion	M5- Expansion	M5+ Percentage	M5 MFI
ND587	S CD3ζ&β2M M5	Marginally decreased vs. KO CD3ζ&β2M M5, equivalent at 1:1 ratio	Increased vs. KO CD3ζ&β2M M5, equivalent at 1:1 ratio	Equivalent to KO CD3ζ&β2M M5	Significantly decreased vs. KO CD3ζ&β2M M5
	KO CD3ζ&β2M M5	Equivalent to M5, decreased only at 1:1 ratio	Marginally increased vs. M5, decreased only at 1:1 ratio	Equivalent to M5	Equivalent to M5
ND610	S CD3ζ&β2M M5	Comparable to KO CD3ζ&β2M M5	Comparable to KO CD3ζ&β2M M5	Marginally decreased vs. KO CD3ζ&β2M M5	Slightly decreased vs. KO CD3ζ&β2M M5
	KO CD3ζ&β2M M5	Decreased vs. M5	Decreased vs. M5	Decreased vs. M5	Decreased vs. M5
ND561	S CD3ζ&β2M M5	Decreased vs. KO CD3ζ&β2M M5	Decreased vs. KO CD3ζ&β2M M5 at 3:1 and 1:1 ratios	Decreased vs. KO CD3ζ&β2M M5	Decreased vs. KO CD3ζ&β2M M5
	KO CD3ζ&β2M M5	Equivalent to or higher than M5	Equivalent to or higher than M5	Equivalent to M5	Equivalent to M5
ND365	S CD3ζ&β2M M5	Slightly lower than KO CD3ζ&β2M M5	Equivalent to or higher than M5	Decreased vs. KO CD3ζ&β2M M5	Quite equivalent to KO CD3ζ&β2M M5
	KO CD3ζ&β2M M5	Decreased vs. M5	Equivalent to or higher than M5	Equivalent to M5	Decreased vs. M5

**ND584**	S CD3ζ&β2M M5	Decreased vs. M5 Decreased vs. KO CD3ζ&β2M M5 (3:1 and 1:1 ratios)	Increased vs. M5 Increased vs. KO CD3ζ&β2M M5 at 10:1 ratio	Decreased vs. KO CD3ζ&β2M M5	Decreased vs. KO CD3ζ&β2M M5
	KO CD3ζ&β2M M5	Decreased vs. M5 at 10:1, higher at 3:1 ratio	Increased vs. M5 at 3:1 and 1:1 ratios	Equivalent to M5	Marginally decreased vs. M5
**ND569**	S CD3ζ&β2M M5	Comparable to KO CD3ζ&β2M M5	Comparable to S CD3ζ&β2M M5	Comparable to KO CD3ζ&β2M M5	Equivalent to KO CD3ζ&β2M M5
	KO CD3ζ&β2M M5	Decreased vs. M5	Decreased vs. M5 at 3:1, higher at 1:1 ratio	Decreased vs. M5	Decreased vs. M5
**ND627**	S CD3ζ&β2M M5	Comparable to KO CD3ζ&β2M M5	Slightly decreased vs. KO CD3ζ&β2M M5 at 1:1 ratio	Comparable to KO CD3ζ&β2M M5	Decreased vs. KO CD3ζ&β2M M5 at 3:1 ratio
	KO CD3ζ&β2M M5	Decreased vs. M5	Decreased vs. M5 except at 1:1 ratio	Decreased vs. M5	Decreased vs. M5 at 1:1 ratio

Supplementary Table 4. Table summarizing the main findings on the expansion rate of both M5CAR+ and M5CAR- T cell populations, percentage of M5CAR+ T cells, and M5CAR MFIs in co-culture with AsPC-1 tumor cells, comparing dual CD3ζ and β2M silenced (S) with dual CD3ζ and β2M knockout (KO) M5CAR T cell groups for each T cell donor (ND587, ND610, ND561, ND365, ND584, ND569, and ND627).

		**% of mice with indicated metastases (1-6)**					
**Group**	**Donor**	**1**	**2**	**3**	**4**	**5**	**6**
**NTD**	**ND584**	n/a	n/a	n/a	n/a	n/a	n/a
	**ND569**	0	16.7	16.7	16.7	83.3	0
	**ND627**	0	0	50	50	75	0
**M5**	**ND584**	0	0	0	0	0	0
	**ND569**	0	0	0	0	0	0
	**ND627**	0	0	0	0	0	0
**S CD3ζ&β2M M5**	**ND584**	28.6	0	0	0	0	0
	**ND569**	0	0	0	0	0	0
	**ND627**	16.7	0	0	0	0	0
**KO CD3ζ&β2M M5**	**ND584**	0	14.3	28.6	0	0	14.3
	**ND569**	0	0	25	25	0	0
	**ND627**	0	0	60	40	0	20

Supplementary Table 5. Improved metastatic control of S CD3ζ&β2M M5CAR T cells relative to KO CD3ζ&β2M M5CAR T cells. In the second *in vivo* mouse experiment, T cell donors ND584, ND569, and ND627 (n= 6-7 mice/group each donor) were used. Mice were euthanized if tumor dimensions exceeded 20 mm, if ulceration covered over 90% of the tumor surface, or if there was a loss in body weight exceeding 20%, according to the approved protocol. A fixed endpoint of 66 days

after CAR T cell injection was set for remaining mice. (**A**) to (**C**) Remaining mice were euthanized on day 66 after CAR T cell injection, and necropsy was performed. The table show the percentage of mice carrying metastases in each treatment group and for each T cell donor. Not transduced T cells, NTD; control M5CAR T cells, M5; dual CD3ζ and β2M silenced (S) M5CAR T cells (S CD3ζ&β2M M5); dual CD3ζ and β2M knockout (KO) M5CAR T cells (KO CD3ζ&β2M M5). Characteristics and tissue distribution of metastases indicated by the numbers (1-6) are as follows: (1) n<2 small metastases in one lung lobe; (2) n>2 small metastases in one lung lobe; (3) n>2 small lung metastases in both lung lobes; (4) large lung metastases; (5) metastases in the same flank as the primary tumor extending towards the upper arm, located in the subcutis outside the peritoneum; (6) spleen metastases. For ND584, n= 0, 6, 7, and 7 mice for NTD, control M5CAR T cell, S CD3ζ&β2M M5CAR T cell, and KO CD3ζ&β2M M5CAR T cell groups, respectively. For ND569, n= 6, 3, 5, and 4 mice for NTD, control M5CAR T cell, S CD3ζ&β2M M5CAR T cell, and KO CD3ζ&β2M M5CAR T cell groups, respectively. For ND627, n= 4, 6, 6, and 5 mice for NTD, control M5CAR T cell, S CD3ζ&β2M M5CAR T cell, and KO CD3ζ&β2M M5CAR T cell groups, respectively.

**First *in vivo* mouse experiment**	**NTD**		**M5**		**S CD3ζ&β2M M5**		**KO CD3ζ&β2M M5**	
**Death causes**	ND587	ND610	ND587	ND610	ND587	ND610	ND587	ND610
End of study		1	4	3	5	3	3	3
End of study with Metastases present								
End of study with GVHD present			1	1				
Euthanised for GVHD			3					
Found dead	1	2		2	2	2	2	
Found dead with GVHD present								
Euthanised for Metastases	6	4			1	1		
Euthanised for Weight Loss > 20% (no Metastases and no GVHD present)	1					2		
Euthanised for Tumor Dimension > 20 mm		1		2	1		3	5
Euthanised for Tumor Dimension > 20 mm with Metastases present								

Supplementary Table 6. Summary of mortality causes in the first *in vivo* mouse experiment. For each treatment group, and considering the different T cell donors, the number of deaths attributed to each listed cause is compiled. Main GVHD symptoms were body weight loss, hunching, reduced activity, loss of fur, diarrhea, and conjunctivitis. N= 8-9 mice/group each donor.

**Second *in vivo* experiment**	**NTD**			**M5**			**S CD3ζ&β2M M5**			**KO CD3ζ&β2M M5**		
**Death causes**	ND 584	ND 569	ND 627	ND 584	ND 569	ND 627	ND 584	ND 569	ND 627	ND 584	ND 569	ND 627
End of study				5		5	5	5	4	3	1	
End of study with Metastases present		6	4				2		1	4	2	5
End of study with GVHD present				1	3	1					1	
Euthanised for GVHD					1							
Found dead	5		1		1							
Found dead with GVHD present					1							
Euthanised for Metastases												
Euthanised for Weight Loss > 20% (no Metastases and no GVHD present)	1		1									
Euthanised for Tumor Dimension > 20 mm								1	1		2	1
Euthanised for Tumor Dimension > 20 mm with Metastases present												

Supplementary Table 7. Summary of mortality causes in the second *in vivo* mouse experiment. For each treatment group, and considering the different T cell donors, the number of deaths attributed to each listed cause is compiled. Main GVHD symptoms were body weight loss, hunching, reduced activity, loss of fur, diarrhea, and conjunctivitis. N= 6-7 mice/group each donor.
